# Seizure outcomes in patients with brain metastases and epilepsy: a systematic review on the efficacy of antitumor treatment and antiseizure medication

**DOI:** 10.1093/nop/npae103

**Published:** 2024-10-22

**Authors:** Josien C C Scheepens, Pim B van der Meer, Linda Dirven, Maaike J Vos, Martin J B Taphoorn, Johan A F Koekkoek

**Affiliations:** Department of Neurology, Leiden University Medical Center, Leiden, the Netherlands; Department of Neurology, Leiden University Medical Center, Leiden, the Netherlands; Department of Neurology, Leiden University Medical Center, Leiden, the Netherlands; Department of Neurology, Haaglanden Medical Center, The Hague, the Netherlands; Department of Neurology, Leiden University Medical Center, Leiden, the Netherlands; Department of Neurology, Leiden University Medical Center, Leiden, the Netherlands; Department of Neurology, Haaglanden Medical Center, The Hague, the Netherlands; Department of Neurology, Leiden University Medical Center, Leiden, the Netherlands

**Keywords:** antiseizure medication, antitumor treatment, brain metastasis, epilepsy, seizure outcomes

## Abstract

**Background:**

Epilepsy is a common symptom in patients with brain metastases (BMs), and because of the rising incidence of BMs, adequate seizure management is warranted. We conducted a systematic review on seizure outcomes after antitumor treatment and antiseizure medication (ASM) in patients with BMs from solid tumors and epilepsy.

**Methods:**

A literature search was performed in 6 databases up to February 2024. Extracted outcomes were rates for (1) seizure freedom, (2) ≥50% seizure reduction, and (3) treatment failure (for ASM only). Weighted averages (WAs) were calculated for outcomes after surgery at 6 months follow-up. Quality assessment of the included studies was performed using the Risk Of Bias In Non-randomized Studies of Interventions (ROBINS-I) tool.

**Results:**

We retrieved 2244 references, of which 16 studies were eligible for inclusion. Eight studies were at critical, and 8 studies at serious risk of bias. The WA of seizure freedom rates at 6 months after surgical resection was 64% (based on 3 studies at serious risk of bias, *n* = 151 patients). Results on ASM efficacy and tolerability were unreliable, as all eligible studies for these outcomes were at critical risk of bias.

**Conclusions:**

Limited available evidence from heterogeneous study populations demonstrated that in the majority of patients with epilepsy due to BMs, seizure freedom 6 months after surgical resection may be reached. No substantial evidence on ASM efficacy and tolerability in patients with epilepsy due to BMs is available. High-quality cohort studies are warranted to expand the evidence on seizure outcomes after antitumor and ASM treatment.

**Key Points**

- Evidence on seizure outcomes in patients with epilepsy due to BMs is at high risk of bias.
- Most patients with epilepsy due to BMs become seizure free 6 months after surgical resection.
- Evidence on the efficacy and tolerability of ASM in patients with epilepsy due to BMs is unreliable.

Brain metastases (BMs) are the most common type of malignant brain tumors. The estimated incidence rate of BMs is 24 per 100,000.^1^ Lung cancer, breast cancer, and melanoma are the most common primary tumors that metastasize to the brain and account for 67%–80% of BMs.^1,2^ The exact incidence proportion of BMs after diagnosis of the primary tumor is difficult to measure but is estimated to add up to 40%–50% during the course of the disease.^3^ Due to better imaging and screening modalities and advancements in systemic treatment, the incidence of BMs has been rising. The use of combination immunotherapy and targeted agents as first-line treatment in several types of cancer has resulted in improved survival outcomes of cancer patients both with and without BMs.^4–6^ For example, patients with BMs from non-small-cell lung cancer (NSCLC) have a significantly better 5-year intracranial progression-free survival after nivolumab plus ipilimumab (16%) than after chemotherapy (6%).^4^ In patients with BMs from melanoma, a patient group that used to have a relatively poor prognosis, 3-year intracranial progression-free survival after nivolumab plus ipilimumab in asymptomatic and symptomatic patients is 54% and 19%, respectively.^7^ Surgical resection or stereotactic radiotherapy could augment the effect of nivolumab plus ipilimumab in patients with BMs from melanoma.^8^ Targeted therapies in patients with molecular subgroups of NSCLC, breast cancer, and melanoma have yielded an impressive improvement in the outcomes of patients with and without BMs.^9^ In NSCLC patients with EGFR mutations and BMs, EGFR-targeting tyrosine kinase inhibitors have resulted in intracranial response rates up to 70%–88%.^10^ Also, ALK-targeting therapies have shown promising results in patients with ALK-rearranged NSCLC and BMs, with intracranial response rates of 78% and 83% with brigatinib and alectinib, respectively.^10–12^

The growing cumulative incidence of BMs is accompanied by an increased risk of developing neurological complications. Main groups of neurological symptoms associated with BMs include focal deficits (eg, motor disorders, hemiparesis, ataxia), signs of increased intracranial pressure (headache, or nausea and emesis), cognitive impairment, and epileptic seizures (generalized or focal). In this systematic review, we will refer to epileptic seizures as “seizures.” The most prevalent symptoms at BM diagnosis are motor disorders (30%), followed by headache (25%), seizures (14%–17%), and nausea and emesis (14%).^13,14^ Epilepsy is more specific for the presence of BMs than any other neurological symptom in patients with breast cancer.^15^ Another 10%–11% of patients develop brain tumor-related epilepsy (BTRE) after BM diagnosis, with an elevated risk of seizures in patients with BMs from melanoma or supratentorial BM location.^16^ Due to the increased survival of patients with BMs, adequate seizure management is becoming of greater importance.

Antitumor treatment may not only prolong overall survival time of patients with a brain tumor but may also reduce neurological symptoms, such as seizures. The choice of antitumor treatment, including surgical resection, radiotherapy, chemotherapy, immunotherapy, and/or targeted therapy, depends on various factors, such as primary tumor type, tumor location, the number of BMs, clinical performance, and overall disease burden.^17,18^ In glioma patients, tumor resection may lead to postoperative seizure freedom, even in patients with drug resistant epilepsy before surgical resection.^19^ In addition, improved seizure control is observed after treatment with radiotherapy or chemotherapy in patients with low-grade glioma. Patients with an objective radiological response more often have seizure reduction, and many patients with stable disease report seizure reduction as well.^20^ There is lack of information on the impact of antitumor treatment on seizure outcomes in patients with BMs.^21^.

Given the high risk of seizure relapse, the occurrence of a single seizure in patients with BMs should be regarded as epilepsy, requiring treatment with ASM according to the current guidelines.^17,22^ There is insufficient evidence to recommend prescribing ASM to reduce the risk of epilepsy in brain tumor patients who have not had an epileptic seizure (ie, ASM as primary prophylaxis), even in the perioperative period.^22^ Epilepsy poses a burden on patients’ health-related quality of life (HRQoL) and is associated with increased morbidity and mortality, particularly in case of uncontrolled seizures.^23,24^ In addition, it has a psychosocial impact on day-to-day functioning, for example, due to driving or employment restrictions, or by causing anxiety and/or depressive symptoms.^25^ Aspects of HRQoL may be significantly impaired by the presence, type, and duration of ASM as well.^26^ In general, patients with a brain tumor may be more susceptible to adverse effects from ASM compared to those with non-brain tumor-related epilepsy, due to complicating factors such as interactions with co-medication (eg, chemotherapy, corticosteroids), exposure to radiotherapy, tumor-related symptoms (eg, headache, nausea), and comorbid psychiatric disorder(s).^27^ A substantial part (33%–50%) of brain tumor patients need to change initial ASM monotherapy due to uncontrolled seizures or intolerable adverse effects, although this evidence is based on studies limited to patients with primary malignant brain tumors or studies that include only a small number of BM patients.^28–30^

Overall, our knowledge on the effect of antitumor or ASM treatment on seizure outcomes in patients with BMs is limited. Increased knowledge on the course of epilepsy after treatment, which has become even more relevant due to the longer survival time of patients with BMs, could help guide clinical decision-making in this patient population. Therefore, we conducted a systematic review on seizure outcomes after antitumor treatment or ASM in patients with BMs from solid tumors and epilepsy.

## Methods

### Search Strategy and Selection Criteria

We performed a literature search in the databases PubMed/MEDLINE, EMBASE, Web of Science, Cochrane Library, Emcare, and PsychInfo up to January 11, 2023 and updated the search on February 12, 2024 to include any newly published studies. Both published studies and conference abstracts were eligible. PRISMA (Preferred Reporting Items for Systematic Reviews and Meta-Analyses) guidelines were followed for screening and selecting references, of which a checklist is provided in Supplementary Material 5. The review was not registered and no review protocol was prepared.

The search strategy included search terms related to (1a) “brain metastasis” or (1b) “brain tumor complications” or (1c) “primary brain tumor,” (2) “epilepsy,” and (3) “outcome.” The PubMed search strategy is depicted in full in Supplementary Material 1. Although this systematic review focused on brain metastases, “primary brain tumor” was also included in the search strategy to prevent missing relevant studies, as this term is placed higher in the MeSH hierarchy in PubMed than “brain metastases.” Two authors (J.C.C.S. and J.A.F.K.) independently assessed which studies were eligible for inclusion based on title and abstract. Afterwards, they reviewed all eligible full-text studies. From the selected studies, reference lists were searched manually for additional sources. Review studies were used to retrieve additional primary research studies for inclusion. Inclusion criteria were (1) adult patients with BTRE due to BMs, (2) antitumor treatment (surgical resection, radiotherapy [ie, whole-brain radiotherapy, focal fractionated irradiation, or stereotactic radiotherapy], or systemic therapy [ie, chemotherapy, immunotherapy, or targeted therapy]) or ASM treatment, (3) a sample size ≥10, (4) seizure outcomes separately reported for an identifiable subset of BM patients or, in case of no identifiable subset, ≥50% of the study population with BMs, (5) published in English, Dutch, or German language in a peer-reviewed journal. Exclusion criteria were (1) no full text available and (2) review published before 2015.

From the included studies, we extracted data regarding study design, sample size, tumor characteristics of the included patients, type of treatment(s) used, follow-up duration, and seizure outcomes.

### Outcomes

Data were collected from the studies by 1 author (J.C.C.S.). In case of any uncertainties, another author (J.A.F.K.) was consulted. In case of antitumor treatment, the following seizure outcomes were assessed: (1) seizure freedom, including Engel class I and International League Against Epilepsy (ILAE) class I; (2) ≥50% reduction in seizure frequency. If available, the radiological response in relation to seizure outcome was reported, which was based on the Response Assessment in Neuro-Oncology brain metastases (RANO-BM) criteria.^31^ In case of ASM, efficacy, defined as the ability to achieve seizure freedom, and effectiveness, which is a combination of efficacy and tolerability (ie, the incidence, severity, and impact of ASM-related adverse events), were the endpoints of interest. Therefore, 3 different outcomes were extracted, if available: (1) seizure freedom, including Engel class I and ILAE class I; (2) ≥50% reduction in seizure frequency; and (3) treatment failure, defined as: (a) discontinuation of the initiated ASM or the need to add-on a second ASM due to uncontrolled seizures, or (b) intolerable adverse effects that resulted in ASM discontinuation. If possible, a weighted average (WA) of each seizure outcome was calculated as follows (to account for differences in sample sizes): the proportion of patients with a certain seizure outcome from each study was multiplied by a weight factor, which is the number of patients included in that study divided by the total number of patients. The sum of the resulting numbers was the WA.

### Quality Assessment

The methodological strength of the included studies was assessed using the Risk Of Bias In Non-randomized Studies of Interventions (ROBINS-I) tool.^32^ This tool is developed to systematically assess the strength of available evidence from non-randomized studies based on seven domains of potential bias. Based on a document providing detailed guidance on how to use the ROBINS-I-tool,^33^ the assessment followed 6 steps for each study included in the systematic review: (1) to formulate the research question based on a hypothetical target trial; (2) to specify the effect of interest: assignment to or starting and adhering to the interventions; (3) to examine how confounders and co-interventions were addressed; (4) to use signaling questions to assess the risk of bias for each of the 7 domains; (5) to provide an overall judgement of the risk of bias for each domain; and (6) to judge the overall risk of bias for the outcome and result being assessed (low risk of bias, moderate risk of bias, serious risk of bias, critical risk of bias, or no information on which to base a judgment about risk of bias). The assessment was based on the available seizure outcomes of interest in the included studies. The ROBINS-I-tool prescribed that the overall risk of bias should at least correspond to the highest level of bias as judged for the 7 domains. It also states that authors may consider risks to be additive, for example, thereby judging a study with serious risk of bias in multiple domains to be at critical overall risk of bias. For this systematic review, as the number of included studies was limited, we decided the overall risk of bias to be conform the highest risk judged for the individual domains. The ROBINS-I-tool stated that studies at a critical risk of bias are “too problematic to provide any useful evidence and should not be included in any synthesis.”^33[p18]^ Therefore, in the results sections on seizure outcomes after antitumor treatment and ASM, we focused on those studies which were not at critical risk of bias.

## Results

**Figure 1:**
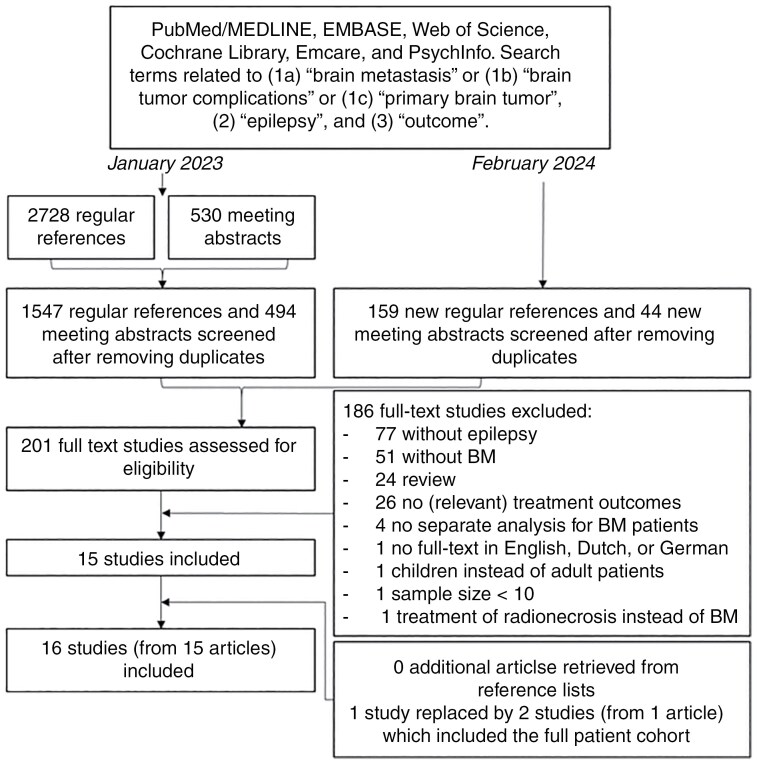
Flowchart of study inclusion.

Up to February 2024, the search identified 1706 unique regular references and 538 meeting abstracts. After screening these abstracts, 201 studies were considered potentially eligible, which were all regular references. The full texts of these studies were assessed, which yielded 15 studies eligible for inclusion. One of the initially selected studies^34^ involved a subset of melanoma patients from 2 large cohorts of BM patients, which were included in full in 2 studies described in one article elsewhere. The studies including the full cohort could be found online and were included in this systematic review instead of the initially selected study, which resulted in a total of 16 studies included in this systematic review.^35^ An overview of the study inclusion process is depicted in Figure 1. We organized the content of the included studies in Table 1, providing an overview of seizure outcomes after antitumor treatment, and Table 2, providing an overview of seizure outcomes after ASM. The results of the quality assessment are presented in Table 3 and the overall risk of bias has been included in Tables 1 and 2. A detailed overview of the quality assessment is provided in Supplementary Material 2.

**Table 1.** Seizure Outcomes After Antitumor Treatment

Study *Risk of bia*s^◦^	Study design	Sample size (with BTRE^*^)	Primary tumor site (with BTRE^*,#^)	Number of BMs	Treatment	Additional treatment, *n* (%)	Follow-up /time of seizure assessment	Seizure outcome (% of total with BTRE*)
Bahna 2022 *Critical*	Retrospective observational cohort study, longitudinal	*N* = 38 (*n* = 38)	• Lung *n* = 16 (*n* = 16) • Breast *n* = 5 (*n* = 5) • Melanoma *n* = 4 (*n* = 4) • Other *n* = 13 (*n* = 13)	• Single: *n* = 30 • Multiple: *n* = 8	Surgical resection	• Levetiracetam *n* = 37 (97%) • Valproic acid *n* = 1 (3%)	At 3 months postoperatively	• Seizure freedom (ILAE I): *n* = 34 (90%) *• ILAE II-IV: *n* = 4 (10%)*
Garcia 2022 *Serious*	Retrospective observational cohort study, longitudinal	*n* = 348 (*n* = 84)	• NSCLC *n* = 118 • Melanoma *n* = 92 • Breast *n* = 82 • Gastrointestinal *n* = 28 • Gynecological *n* = 12 • Renal *n* = 13 • Urothelial *n* = 12	Mean *n* = 3 ± 2.7	Surgical resection	*Preoperative* • ASM *n* = 209 (60%; 100% of patients with BTRE*) • Radiotherapy *n* = 80 (23%) *Postoperative* • ASM *n* = 149 (43%; 100% of patients with BTRE*) • Radiotherapy *n* = 225 (65%)	At 6 months postoperatively	• Seizure freedom (Engel class I): *n* = 48 (57%) *• Engel class II: *n* = 16 (19%) *• Engel class III: n = 12* (14%) • Engel class IV: *n* = 8 (10%)*
Puri 2020 *Critical*	Retrospective observational cohort study, longitudinal	*n* = 286 (*n* = 48)	• Lung *n* = 107 (*n* = 22) • Breast *n* = 37 (*n* = 8) • Gastrointestinal *n* = 25 (*n* = 2) • Skin *n* = 42 (*n* = 2) • Renal *n* = 10 (*n* = 1) • Reproductive *n* = 10 (*n* = 0); • Other/unknown *n* = 55 (*n* = 8)	• Single: *n* = 237 • Multiple: *n* = 49	Surgical resection	Not specified	At 3 months postoperatively	• Seizure freedom: *n* = 36 (75%) *• Any seizure activity: *n* = 12 (25%)*
Wu 2017 *Serious*	Retrospective observational cohort study, longitudinal	*n* = 565 (*n* = 114)	• NSCLC *n* = 213 (*n* = 52) • SCLC *n* = 25 • Breast *n* = 80 (*n* = 18) • Gastrointestinal *n* = 50 (*n* = 7) • Skin *n* = 81 (*n* = 12) • Renal *n* = 41 (*n* = 8) • Other *n* = 75 (*n* = 17)	Mean *n* = 1.73	Surgical resection	*Preoperative* *• *Chemotherapy *n* = 294 (52%) *Postoperative* • ASM *n* = 110 (97% of patients with BTRE*) • Chemotherapy *n* = 198 (35%) • WBRT *n* = 270 (48%) • SRT *n* = 186 (33%)	At 1, 3, 6, 12, 24 months postoperatively	Seizure freedom (Engel class I) • At 1 month: *n* = 76 (94%) • At 3 months: *n* = 52 (88%) • At 6 months: *n* = 36 (88%) • At 12 months: *n* = 23 (85%) *• *At 24 months: *n* = 13 (93%)
Sanmillan 2017 *Critical*	Retrospective observational cohort study, longitudinal	*n* = 33 (*n* = 13)	• Lung *n* = 17 • Breast *n* = 7 • Kidney *n* = 5 • Other *n* = 4	Single: *n* = 33	Surgical resection	*Preoperative* • ASM *n* = 13 (100% of patients with BTRE*) *Postoperative* • ASM *n* = 14 (100% of patients with BTRE*) • WBRT: *n* = 28 (85%)	At 6 months postoperatively	• Seizure freedom: *n* = 13 (100%)
Pelletier 2021 *Serious*	Retrospective observational case-control study	*n* = 20 (*n* = 10)	• Lung *n* = 10 • Breast *n* = 4 • Melanoma *n* = 2 • Gastrointestinal *n* = 1 • Kidney *n* = 1 • Perivascular epithelioid cell *n* = 1 • Testis non seminomatous germ cell *n* = 1.	Single: *n* = 20	Surgical resection	*Preoperative* • Radiotherapy *n* = 10 (50%) • Chemotherapy *n* = 12 (60%)	At 3 months postoperatively	• Controlled seizures**: n = 9 (90%) ◦ Seizure reduction**: n = 3 (30%) ◦ No change in seizure control n = 6 (60%)
Huntoon 2023 *Critical*	Retrospective observational cohort study, longitudinal	*n* = 1581 (*n* = 136)^##^	• Melanoma *n* = 222 (*n* = 44) • NSCLC *n* = 863 (*n* = 55) • Breast *n* = 325 (*n* = 22) • Colon *n* = 62 (*n* = 6) • Renal cell *n* = 109 (*n* = 9)	*With BTRE* • Single: *n* = 49 • Multiple: *n* = 87	Surgical resection vs. no surgical resection	Not specified	At last follow-up, ≥ 2 years	*After surgical resection* • Seizure freedom *n* = 48 (60%) • ≥ 50% seizure reduction *n* = 55 (68.8%) *After no surgical resection* • Seizure freedom *n* = 27 (48.2%) • ≥ 50% seizure reduction *n* = 34 (62.5%)
Cummins 2023 *Serious*	Retrospective observational cohort study, longitudinal	*n* = 112 (*n* = 26)	• Melanoma *n* = 35 • NSCLC *n* = 32 • Breast *n* = 13 • Gastrointestinal *n* = 9 • Renal cell *n* = 6 • Gynecological *n* = 5 • Other *n* = 14	Median *n* = 2, range 1-25	Surgical resection	*Preoperative* *• *ASM *n* = 26 (100% of patients with BTRE*) *• *Radiotherapy *n* = 30 (26%) *Postoperative* • ASM *n* = 112 (100%)^ • Brachytherapy *n* = 10 (9%) • SRT *n* = 79 (69%) *• *WBRT *n* = 3 (3%) *• *Immune checkpoint inhibitor *n* = 40 (35%) *• *Targeted therapy *n* = 44 (39%)	At 6 months postoperatively	• Seizure freedom (Engel class I): *n* = 13 (50%) *• Engel class II: n = 6 (23%)* *• Engel class III: n = 5 (19%)* • *Engel class IV: n = 2 (8%)*
Lee 2013 *Critical*	Retrospective observational cohort study, longitudinal	*n* = 258 (*n* = 32)	• NSCLC *n* = 224 (*n* = 25) • SCLC *n* = 20 (*n* = 5) • Unknown *n* = 14 (*n* = 2)	Mean *n* = 3.5 with seizures (*n* = 2.9 without seizures)	GKS	*Before GKS* • WBRT: *n* = 26 (10%) *After GKS* • WBRT: *n* = 75 (29%) • Craniotomy^$^: *n* = 17 (7%) *Before or after GKS* • ASM: *n* = 75 (100% of patients with BTRE*)	At last follow-up, median 69 weeks (range 1–254) after BM diagnosis	• Seizure freedom without ASM: *n* = 14 (44%) • Seizure freedom with ASM: *n* = 4 (13%) *• Seizures despite ASM: n = 5 (16%)*
Borgelt 1980, study I *Serious*	*Primary outcomes* • RCT *Seizure outcomes* *• *Experimental intervention study without control group&	*n* = 910 (*n* = 193)^^	• Lung *n* = 560 • Breast *n* = 166 • Melanoma *n* = 29 • Colon *n* = 25 • Kidney *n* = 15 • Other *n* = 47 • Unknown *n* = 68	Not specified	WBRT in different time-dose schedules per group	*During WBRT* • Corticosteroids: *n* = 637 (70%) • Chemotherapy *n* = 164 (18%)	Follow-up until death since WBRT initiation, median 18 weeks	• Seizure freedom: *n* = 120 (62%) • Seizure reduction**: *n* = 158 (82%)^^
Borgelt 1980, study II *Serious*	*Primary outcomes* • RCT *Seizure outcomes* Experimental intervention study without control group&	*n* = 902 (*n* = 134)	• Lung *n* = 507 • Breast *n* = 146 • Melanoma *n* = 28 • Colon *n* = 27 • Kidney *n* = 24 • Other *n* = 82 Unknown *n* = 88	Not specified	WBRT in different time-dose schedules per group	*During WBRT* • Corticosteroids *n* = 622 (69%) Chemotherapy *n* = 198 (22%)	Follow-up until death since WBRT initiation, median 15 weeks	• Seizure freedom: *n* = 117 (87%) • Seizure reduction**: n = 120 (90%)
Miller 2023 *Serious*	Retrospective observational cohort study, longitudinal	*Total inclusion* *n* = 444 (*n* = 44) *Inclusion to seizure analysis* *n* = 288 (*n* = 34)	*Inclusion to seizure analysis* • NSCLC *n* = 105 • Breast *n* = 56 • Melanoma *n* = 39 • Renal cell *n* = 21 • Head and neck *n* = 15 • Gastrointestinal *n* = 14 • Gynecological *n* = 11 • Other *n* = 27	Median *n* = 2, interquartile range 1, 4	SRS^&&^	*Total inclusion* *Before SRS* • Surgery: *n* = 104 (23%) *Inclusion to seizure analysis* *Before SRS* *• *ASM: *n* = 29 (85% of patients with BTRE*)	At 3 months after SRS	• Seizure freedom: *n* = 26 (76%)
YZ Kim 2011 *Serious*	Prospective observational cohort study, longitudinal	*n* = 103 (*n* = 36)	• NSCLC *n* = 67 • SCLC *n* = 6 • Renal cell *n* = 9 • Hepatocellular *n* = 7 • Colorectal *n* = 5 • Breast *n* = 3 • Other *n* = 6	Single: *n* = 103	Chemo-therapy + topiramate^$^	In patients with BTRE* Before chemotherapy • Surgical resection *n* = 20 (56%) • Radiosurgery *n* = 10 (28%) • WBRT *n* = 2 (5.6%) • SRT *n* = 1 (2.8%)	Median 8.7 months (during chemotherapy cycle)	• Seizure freedom *n* = 32 (89%)

ASM = antiseizure medication; BM = brain metastasis; BTRE = brain tumor-related epilepsy; GKS = gamma knife radiosurgery; ILAE = International League Against Epilepsy; NSCLC = non-small-cell lung cancer; RCT = randomized controlled trial; SCLC = small cell lung cancer; SRS = stereotactic radiosurgery; SRT = stereotactic radiotherapy; WBRT = whole brain radiotherapy.

^◦^Risk of bias as assessed using the Risk Of Bias In Non-randomized Studies of Interventions (ROBINS-I) tool

^*^BTRE before the initiation of the antitumor treatment of study;

^#^If provided in the article;

^**^Definition unclear;

^##^Referring to the patients included in the final analysis;

^^^ASM prophylaxis was typically given for 30 days;

^$^Definition (surgical resection of BM or else) unclear.

^&^Seizure outcomes were presented for all included patients after WBRT as one group. Therefore, the part of the study on seizure outcomes is considered an experimental intervention study without a control group;

^^^^The numbers presented here were derived from adding up numbers of the 2 groups presented in the article: one with focal seizures and one with generalized seizures;

^&&^Single-fraction SRS, hypofractionated SRS or a combination;

^$$^Started in all patients (with and without seizures) 2 weeks before chemotherapy, the dose schedule was standardized.

**Table 2.** Efficacy and Tolerability of ASM

Study *Risk of bias*◦	Study design	Sample size*	Primary tumor site	Number of BM	Treatment	Additional treatment, n (%)	Follow-up / time of seizure assessment	Seizure outcomes, n (%)
Newton 2007 *Critical*	Retrospective observational cohort study, longitudinal	*n* = 13	• Breast *n* = 6 • Lung *n* = 5 • Melanoma *n* = 2	Not specified	*ASM* • Levetiracetam monotherapy: *n* = 6 (46%) • Levetiracetam as add-on: *n* = 7 (54%)	*Before ASM* • Radiotherapy: *n*=“majority” *Before or during ASM* • Chemotherapy: *n*=not specified	At 1 month after start ASM	• Seizure freedom *n* = 10 (77%). • ≥ 50% seizure reduction: *n* = 13 (100%) • Treatment failure: *n* = 0 (0%)
Maschio 2022 *Critical*	Retrospective observational cohort study, longitudinal	*n* = 42	• Lung *n* = 21 • Breast *n* = 13 • Melanoma *n* = 4 • Bladder *n* = 2 • Other *n* = 2	Median *n* = 1, range 1–8	*ASM* m*onotherapy or polytherapy* • Levetiracetam: *n* = 26 (62%) • Oxcarbazepine: *n* = 4 (10%) • Phenobarbital: *n* = 4 (10%) • Other: *n* = 8 (19%)	*At diagnosis* • Surgical resection: *n* = 18 (43%) • Radiotherapy: *n* = 37 (88%) • Systemic therapy: *n* = 35 (83%)	At last follow-up, median 11.4 months after BM diagnosis	*At last follow-up* • Seizure freedom**: *n* = 24 (57%). • Treatment failure: *n* = 21 (50%) *At 7 months* • Treatment failure: *n* = 19 (45%)
Maschio 2010 *Critical*	Prospective observational cohort study, longitudinal	*n* = 30	• Lung *n* = 15 • Melanoma *n* = 5 • Breast *n* = 5 • Colon *n* = 2 • Thyroid *n* = 1 • Mediastinum *n* = 1 • Prostate *n* = 1.	• Single: *n* = 19 • 2 BM: *n* = 3 • ≥3 BM: *n* = 8	*ASM monotherapy* • Levetiracetam: *n* = 6 (20%) • Oxcarbazepine *n* = 16 (53%) • Topiramate: *n* = 8 (27%)	*Before ASM* • Surgical resection: *n* = 8 • Chemotherapy and/or radiotherapy: *n* = 23 (77%)	Until death (mean 6.1 months) after first visit	• Seizure freedom: *n* = 19 (63%) • Treatment failure: *n* = 0 (0%) *• Reduction mean seizure frequency/month: 6.5* →*1.3 (P < .001)*

ASM = antiseizure medication; BM = brain metastasis.

^◦^Risk of bias as assessed using the Risk Of Bias In Non-randomized Studies of Interventions (ROBINS-I) tool.

^*^All included patients had seizures at baseline; **Despite any treatment modifications.

**Table 3.** Quality Assessment of the Included Studies Based on the Risk of Bias

Article	Overall bias	Confounding	Selection of patients	Classification of interventions	Deviations from intended interventions	Missing data	Measurement of outcomes	Selection of reported results
Bahna 2022	**Critical**	Critical	Serious	Low	Serious	Moderate	Moderate	Low
Garcia 2022	**Serious**	Serious	Low	Low	Moderate	Serious	Moderate	Low
Puri 2020	**Critical**	Serious	Low	Low	Critical	Serious	Moderate	Low
Wu 2017	**Serious**	Serious	Low	Low	Moderate	Serious	Moderate	Low
Sanmillan 2017	**Critical**	Critical	Low	Low	Moderate	Serious	No information	Low
Pelletier 2021	**Serious**	Serious	Low	Low	Serious	Low	No information	Low
Huntoon 2023	**Critical**	Serious	Serious	Low	Critical	Serious	Moderate	Serious
Cummins 2023	**Serious**	Serious	Serious	Low	Moderate	Serious	Moderate	Low
Lee 2013	**Critical**	Critical	Low	Low	Serious	Serious	Moderate	Low
Borgelt 1980*	**Serious**	Serious	No information	Low	Serious	Serious	Moderate	Moderate
Miller 2023	**Serious**	Serious	Serious	Low	Moderate	Serious	Moderate	Low
Kim 2011	**Serious**	Serious	Low	Low	Moderate	Serious	Moderate	Low
Newton 2007	**Critical**	Critical	Serious	Low	Critical	Low	Moderate	Low
Maschio 2022	**Critical**	Serious	Low	Low	Moderate	Critical	Moderate	Low
Maschio 2010	**Critical**	Serious	Low	Low	Serious	Critical	Moderate	Low

^*^The article by Borgelt et al. (1980) contained 2 studies with identical study designs and at identical risk of bias, which were therefore depicted jointly here.

### Study Characteristics

Sixteen unique studies (which were described in 15 articles) were included in this systematic review. Thirteen studies were observational cohort studies^13,29,36–46^ which measured longitudinally, of which 11 had a retrospective^13,29,36–39,41,43–46^ and 2 a prospective^40,42^ study design. One study had a retrospective observational case–control study design^47^ and 2 studies had an experimental study design, in which seizure outcomes were analyzed as intervention studies without a control group (more details are provided in Table 1).^35^ Thirteen out of 16 reported seizure outcomes after antitumor treatment,^13,35–41,43,45–47^ of which 8 studies discussed outcomes after surgical resection,^13,36–39,45–47^ 1 after Gamma Knife Radiosurgery,^41^ 2 after whole-brain radiotherapy,^35^ 1 after systemic radiosurgery,^43^ and 1 after chemotherapy.^40^ Three studies investigated the effect of different ASMs on seizure outcomes, of which levetiracetam and oxcarbazepine were most commonly prescribed.^29,42,44^ Twelve out of 13 studies on antitumor treatment comprised patients with and without BTRE before the start of the treatment under study,^13,35,37–41,43,45–47^ whereas all 3 studies on ASM included patients with BTRE only.^29,42,44^ Numbers of patients with BTRE included in the studies ranged from *n* = 10^47^ to *n* = 193,^35^ of which only 4 studies had more than *n* = 100 patients included.^35,39,46^ Four studies^13,40,45,47^ included patients with a single BM, 9^29,36–39,41–43,46^ studies with a single BM or multiple BMs, and in 2 studies,^35,44^ the number of BMs was not specified. Patients with BMs from different primary tumor sites were included. Most common were BMs originating from lung and breast cancer and melanoma, as can be expected based on their epidemiology. All studies except 2 reported which additional treatments patients received during the study period, which are depicted in Tables 1 and 2 along with the timing of treatment.^13,39^ Overall, 8 studies were considered at serious risk of bias^35,37,38,40,43,46,47^ and 8 studies at critical risk of bias.^13,29,36,39,41,42,44,45^ All 8 studies at serious risk of bias discussed seizure outcomes after antitumor treatment.

### Seizure Outcomes After Antitumor Treatment

**Figure 2:**
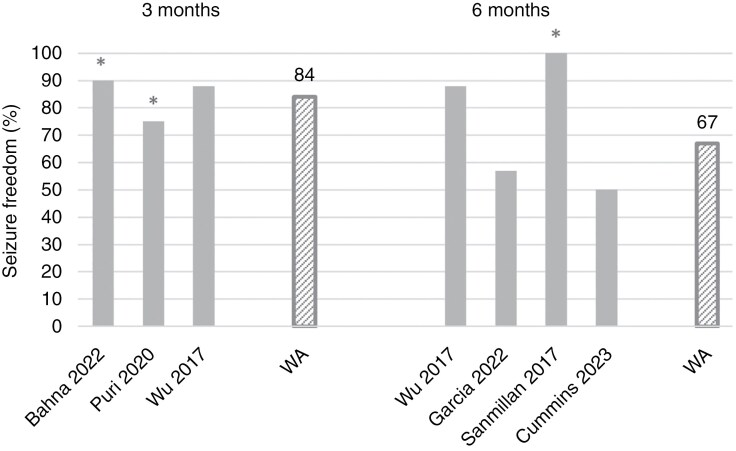
Postoperative seizure freedom. * Studies at critical risk of bias; WA = weighted average.

The key findings of the 13 available studies on seizure outcomes after antitumor treatment are depicted in Table 1. Omitting the studies at critical risk of bias, we included 4 studies on seizure outcomes after surgical resection,^37,38,46,47^ 2 after whole-brain radiotherapy,^35^ 1 after stereotactic radiosurgery,^43^ and 1 study on seizure outcomes after chemotherapy.^40^ Seven studies reported seizure freedom,^35,37,38,40,43,46^ whereas none of the studies reported ≥50% seizure reduction. No studies reported radiological response in relation to seizure outcome based on the RANO-BM criteria. In one study on seizure outcomes after surgical resection, seizure control and seizure reduction were reported but could not be interpreted, as a clear definition of these outcomes was lacking.^47^ Based on the available evidence of the other 3 studies, the WA of seizure freedom after surgical resection at 6 months was 64%.^37,38,46^ If all studies were considered regardless of the risk of bias, WAs of seizure freedom at 3 and 6 months postoperatively could be calculated based on the available evidence from 6 studies.^13,36–38,45,46^ In a total sample size of *n* = 145 and *n* = 164 patients, seizure freedom after surgical resection would be 84% and 67% at 3 and 6 months, respectively. Weighted averages of postoperative seizure freedom including and excluding the studies at critical risk of bias are shown in Figure 2 and in Supplementary Materials 3 and 4. Two large studies included in one article on whole-brain radiotherapy, which was published in 1980, found seizure freedom rates of 62% (*n* = 193) and 87% (*n* = 134) after a median of plus minus 4 months of follow-up.^35^

ASM was the most frequently reported additional treatment used during the study period, which reflects common clinical practice. Additional ASM was prescribed to all patients with and without seizures in 2 studies,^37,40^ and (almost) all patients with epilepsy in the remaining 5 studies received ASM.^37,38,40,43,46^ Especially in the study by Kim et al.^40^ on the effect of chemotherapy on seizure outcomes, part of the antiseizure effect could be contributed to topiramate, as its use was standardized for all patients during the whole chemotherapy treatment period.

### Efficacy of ASM

Table 2 provides an overview of the key findings of the 3 included studies on efficacy of ASM.^29,42,44^ However, after omitting the studies at critical risk of bias, no evidence on seizure outcomes after ASM treatment is available based on this systematic review.

## Discussion

In this systematic review, we investigated the effect of antitumor treatment and ASM on seizure outcomes in patients with epilepsy due to BMs from solid tumors. Of all treatment modalities and seizure outcomes studied, most substantial evidence was available on seizure freedom rates after surgical resection. After exclusion of the studies at critical risk of bias, we found a WA of postoperative seizure freedom of 64% at 6 months. Overall, the evidence was limited in quality, number of studies, and sample sizes. Based on systematic assessment of the risk of bias using the ROBINS-I-tool, 8 studies included in this systematic review were assessed to be at critical overall risk of bias, and 8 studies were at serious overall risk of bias. In general, risk of bias due to confounding, deviations from intended interventions and bias due to missing data were the methodological areas of particular concern. Due to the critical overall risk of bias of the 3 studies on efficacy and tolerability of ASM, we could not draw reliable conclusions on the effectiveness of ASM in patients with BMs and epilepsy. Also, studies including patients with BMs without a history of epilepsy were excluded from this systematic review, and therefore the efficacy of antitumor treatment and ASMs in seizure-naïve BM patients falls outside the scope of this review.

The concerns regarding the quality of the (already limited) evidence included in this systematic review emphasize the need to expand evidence on seizure outcomes in patients with BMs and epilepsy. We suggest that large cohort studies should be conducted, as these have considerable advantages over randomized trials regarding time and cost efficiency and feasibility, while the risk of bias can be limited with solid methodology. The current research sparsity might be due to the lower incidence of seizures in patients with BMs compared to primary brain tumors. The mean prevalence of seizures in different types of glioma is around 60%, ranging from 34% in glioblastoma, isocitrate dehydrogenase-wildtype, up to 94% in dysembryoblastic neuro-epithelial tumors, whereas 23% of patients with BMs develop seizures.^48,49^ Expanding our knowledge on seizure outcomes in BMs has become clinically more relevant, as the number and survival of patients with BMs is increasing, resulting in a higher risk of long-term burden of epilepsy in this patient group. Two decades ago, 5%–20% of patients were reported to develop BMs from lung and breast carcinoma and melanoma, whereas now these tumors are estimated to metastasize to the brain in 40%–50% of patients.^3,50,51^ The longer survival of patients with BMs from solid tumors has further contributed to the rising number of patients living with BMs. For example, 5-year intracranial progression-free survival of patients with BMs from NSCLC almost tripled due to the introduction of combination immunotherapy.^4^ Recent studies suggest a synergistic effect between radiotherapy and immunotherapy, which might further improve the prognosis.^52,53^ However, with the growing number of treatment options for patients with BMs, long-term neurotoxicity, such as radiation necrosis, is also more likely to occur, which might cause or aggravate neurologic symptoms including seizures.^54,55^ Bevacizumab may effectively reduce radiation necrosis after failure of corticosteroids and may contribute to improved seizure control.^56,57^

In general, patients with supratentorial, especially frontal, BMs are at highest risk of developing seizures, as well as younger patients.^16,38,58–60^ Metastases from melanoma have the largest seizure prevalence among BMs, which has been contributed to their tendency to involve the cortex, hemorrhage, and present with multiple lesions.^16,58,61^ Evidence is inconsistent regarding the correlation between number and size of BMs and seizure occurrence. Both single BM and > 4 BMs and smaller as well as larger size of BMs have been alternately identified as risk factors for seizures in different studies.^16,59,60,62,63^ One study included in this systematic review,^41^ although at critical risk of bias, found that seizure occurrence was associated with radiological changes, such as new or progressive BMs, in 78% of cases, which is consistent with previous research associating BM progression with seizures after correcting for confounding variables.^64^ Ambiguous evidence exists regarding extent of resection as a risk factor for seizures. In a cohort of BM and glioma patients (*n* = 650), gross total resection was associated with increased seizure risk (odds ratio [OR] = 15.5, 95% confidence interval [CI] = 2.73–171.6), which the authors contributed to damage-related epileptogenicity in the surrounding brain tissue.^60^ However, in a study including BM patients (*n* = 220), complete resection provided a lower risk of seizures compared to incomplete resection (OR = 0.2, 95% CI = 0.04–0.7).^64^ The latter finding is consistent with earlier literature in glioma patients^65^ and corresponds with the favorable seizure outcomes we found after surgical resection in this systematic review.

The pathophysiology of epilepsy in BMs remains to be elucidated, although some contributing factors have been found so far. Seizures are caused by abnormal electrical activity in the cortex based on neuronal hyperexcitability, which could be provoked by peritumoral brain ischemia. In BMs, microvascular changes reduce peritumoral perfusion pressure and disrupt the tumoral microcirculation, thereby causing ischemia. Mechanical compression, due to the noninfiltrative growth pattern of BMs and the presence of peritumoral edema, also induces ischemia.^58,66,67^ More speculative is the role of neurotransmitter activity in seizure development in BMs. In glioma, increased excitatory glutamate release and decreased inhibitory activity of gamma-aminobutyric acid (GABA) in tumoral and peritumoral tissue, which may be caused by disruption of the blood–brain barrier, are associated with seizures.^58,66,68,69^ The same neurotransmitter imbalance promotes oncogenesis in glioma.^66,70^ In BM oncogenesis, glutamate and GABA also play an important role.^71–73^ In breast cancer, glutamate ligands of n-methyl-d-aspartate receptors activate a neuronal signaling pathway, which induces metastatic colonization of the brain. Metastatic cells produce insufficient glutamate to activate the signaling themselves but instead form synapses between cancer cells and glutamatergic neurons to start this process.^73^ Furthermore, BMs express GABA receptors and metabolize available GABA to spread to the brain and adapt to the neural microenvironment.^71,72^ As far as we know, no studies have investigated the correlation between glutamate and GABA activity and epileptogenesis in BMs yet. One study^74^ found lower levels of glutamate in metastatic cell cultures compared to glioblastoma but did not investigate the epileptogenic properties of those cell lines. Future research may show if the mechanisms involved in oncogenesis and epileptogenesis in BMs overlap similarly as in glioma.

In conclusion, limited available evidence suggests that seizure freedom 6 months postoperatively can be reached in the majority of patients with BMs. Regarding efficacy and tolerability of ASM, current research is inconclusive, as the limited available evidence is of too low quality. In the light of the growing number of patients with seizures due to BMs, large, well-designed cohort studies are needed to guide antitumor and ASM treatment in patients with BMs and seizures.

## Supplementary material

Supplementary material is available online at *Neuro-Oncology Practice* (https://academic.oup.com/nop/).

npae103_suppl_Supplementary_Tables

npae103_suppl_Supplementary_Checklist

## Data Availability

No new data were generated or analyzed in support of this research. All contributions presented in this study are included in the article and the Supplementary materials. Further inquiries can be directed to the corresponding author.
