# Supplementary material for: Seizure outcomes in patients with brain metastases and epilepsy: a systematic review on the efficacy of antitumor treatment and antiseizure medication
Source: Neurooncol Pract. 2024 Oct 22;12(3):376–88. doi: 10.1093/nop/npae103 (PMC12137217; doi:10.1093/nop/npae103)
Supplement: npae103_suppl_Supplementary_Tables [file npae103_suppl_supplementary_tables.docx]

**Supplementary materials**

Supplementary 1: Search strategy PubMed
Supplementary 2: Detailed quality assessment of the Risk Of Bias In Non-randomized Studies of Interventions tool
Supplementary 3: Seizure outcomes at 6 months postoperatively
Supplementary 4: Seizure outcomes at 3 and 6 months postoperatively, including articles at critical overall risk of bias

**Supplementary 1: Search strategy PubMed**

*Components of strategy*a. - brain **metastasis**, epilepsy, outcome

b. - brain tumor **complications**, epilepsy, outcome

c. - primary brain tumor, epilepsy, outcome

*Full strategy***(**(("Brain Neoplasms/**secondary**"[Mesh:noexp] OR **"secondary brain tumor"[tw] OR "secondary brain tumour"[tw] OR "brain metastasis"[tw] OR "brain metastases"[tw] OR "secondary brain tumor"[tw] OR "secondary brain tumour"[tw] OR "brain metastasis"[tw] OR "brain metastases"[tw]** **OR "brain metastatic"[tw]** **OR "brain metasta*"[tw]** **OR** **"distant recurrence of brain"[tw] OR "distant recurrences of brain"[tw] OR "recurrent cancer of brain"[tw] OR "brain recurrence"[tw] OR "recurrent cancers of brain"[tw] OR "brain recurrences"[tw]** OR **"distant recurrence of brain"[tw] OR "distant recurrences of brain"[tw] OR "recurrent cancer of brain"[tw] OR "brain recurrence"[tw] OR "recurrent cancers of brain"[tw] OR "brain recurrences"[tw]** OR **"brain metastasis"[title/abstract:~3] OR "brain metastases"[title/abstract:~3] OR "brain metastatic"[title/abstract:~3] OR "brain secondary"[title/abstract:~3] OR "cns metastasis"[title/abstract:~3] OR "cns metastases"[title/abstract:~3] OR "cns metastatic"[title/abstract:~3] OR "cns secondary"[title/abstract:~3] OR "central nervous metastasis"[title/abstract:~3] OR "central nervous metastases"[title/abstract:~3] OR "central nervous metastatic"[title/abstract:~3] OR "central nervous secondary"[title/abstract:~3] OR "cerebral metastasis"[title/abstract:~3] OR "cerebral metastases"[title/abstract:~3] OR "cerebral metastatic"[title/abstract:~3] OR "cerebral secondary"[title/abstract:~3] OR "intracerebral metastasis"[title/abstract:~3] OR "intracerebral metastases"[title/abstract:~3] OR "intracerebral metastatic"[title/abstract:~3] OR "intracerebral secondary"[title/abstract:~3] OR "cranial metastasis"[title/abstract:~3] OR "cranial metastases"[title/abstract:~3] OR "cranial metastatic"[title/abstract:~3] OR "cranial secondary"[title/abstract:~3] OR "brain metastasis"[title/abstract:~3] OR "brain metastases"[title/abstract:~3] OR "brain metastatic"[title/abstract:~3] OR "brain secondary"[title/abstract:~3]** OR (("Brain Neoplasms"[Mesh:noexp] OR "Brain tumor"[tw] OR "brain tumors"[tw] OR "brain tumour"[tw] OR "brain tumours"[tw] OR "brain neoplasm"[tw] OR "brain neoplasms"[tw] OR "central nervous system cancer"[tw] OR "cns cancer"[tw] OR "cns cancers"[tw] OR "cerebral cancer*"[tw] OR "intracerebral cancer*"[tw] OR "intracranial cancer*"[tw] OR "cranial cancer*"[tw] OR "central nervous system tumor*"[tw] OR "central nervous system tumour"[tw] OR "tumor of the central nervous system"[tw] OR "tumour of the central nervous system"[tw] OR "cns tumor"[tw] OR "cns tumors"[tw] OR "cns tumour"[tw] OR "cns tumours"[tw] OR "cerebral tumor"[tw] OR "cerebral tumour"[tw] OR "cerebral tumors"[tw] OR "cerebral tumours"[tw] OR "intracerebral tumor"[tw] OR "intracerebral tumour"[tw] OR "intracerebral tumors"[tw] OR "intracerebral tumours"[tw] OR "intracranial tumor"[tw] OR "intracranial tumour"[tw] OR "intracranial tumors"[tw] OR "intracranial tumours"[tw] OR "cranial tumor"[tw] OR "cranial tumour"[tw] OR "cranial tumors"[tw] OR "cranial tumours"[tw] OR "brain malignancy"[tw] OR "brain malignancies"[tw] OR "cns malignancy"[tw] OR "malignancy of the central nervous system"[Title/Abstract:~3] OR "malignancies of the central nervous system"[Title/Abstract:~3] OR "malignant central nervous system"[Title/Abstract:~3] OR "malignancy central nervous system"[Title/Abstract:~3] OR "malignancies central nervous system"[Title/Abstract:~3] OR "malignant central nervous system"[Title/Abstract:~3] OR "central nervous system malignancy"[tw] OR "central nervous system malignancies"[tw] OR "cerebral malignancy"[tw] OR "cerebral malignancies"[tw] OR "intracerebral malignancy"[tw] OR "intracerebral malignancies"[tw] OR "intracranial malignancy"[tw] OR "intracranial malignancies"[tw] OR "cranial malignancy"[tw] OR "cranial malignancies"[tw] OR "brain carcinoma"[tw] OR "carcinoma of the brain"[tw] OR "brain carcinomas"[tw] OR "cns carcinoma"[tw] OR "cns carcinomas"[tw] OR "carcinoma of the central nervous system"[Title/Abstract:~3] OR "carcinomas of the central nervous system"[Title/Abstract:~3] OR "carcinoma central nervous system"[Title/Abstract:~3] OR "carcinomas central nervous system"[Title/Abstract:~3] OR "central nervous system carcinoma"[tw] OR "central nervous system carcinomas"[tw] OR "cerebral carcinoma"[tw] OR "cerebral carcinomas"[tw] OR "intracerebral carcinoma"[tw] OR "intracerebral carcinomas"[tw] OR "intracranial carcinoma"[tw] OR "intracranial carcinomas"[tw] OR "cranial carcinoma"[tw] OR "cranial carcinomas"[tw]) AND **("Neoplasm Metastasis"[Mesh] OR "metastasis"[tw] OR "metastatic"[tw] OR "metasta*"[tw] OR "secondary"[tw])**)) AND ("Epilepsy"[Mesh] OR "Epilepsy"[tw] OR "epileptic"[tw] OR "epilepsia"[tw] OR "epilep*"[tw] OR "Seizures"[Mesh] OR "seizure"[tw] OR "seizures"[tw] OR "Epilepticus"[tw] OR "insult"[tw] OR "insults"[tw] OR "convulsion"[tw] OR "convulsions"[tw] OR "Anticonvulsants"[Mesh] OR "Anticonvulsants"[Pharmacological Action] OR "Anticonvulsants"[tw] OR "Anticonvulsant"[tw] OR "Antiepileptic"[tw] OR "Antiepileptic"[tw] OR "Antiseizure"[tw] OR "Antiseizures"[tw] OR "1,3-ditolylguanidine"[Supplementary Concept] OR "1-(4-chlorophenyl)-4-piperidin-1-yl-1,5-dihydroimidazol-2-one"[Supplementary Concept] OR "2,3-dioxo-6-nitro-7-sulfamoylbenzo(f)quinoxaline"[Supplementary Concept] OR "2,3-piperidinedicarboxylic acid"[Supplementary Concept] OR "2-(2,3-dicarboxycyclopropyl)glycine"[Supplementary Concept] OR "2-amino-4-methyl-5-phosphono-3-pentenoic acid"[Supplementary Concept] OR "2-amino-4-phosphonobutyric acid"[Supplementary Concept] OR "2-amino-7-phosphonoheptanoic acid"[Supplementary Concept] OR "2-fluoro-2-phenyl-1,3-propanediyl dicarbamate"[Supplementary Concept] OR "2-propyl-2-pentenoic acid"[Supplementary Concept] OR "3-(2-carboxypiperazin-4-yl)propyl-1-phosphonic acid"[Supplementary Concept] OR "4-amino-3-phenylbutyric acid"[Supplementary Concept] OR "4-phenyl-perhydropyrrole(1,2-a)pyrazine-1,3-dione"[Supplementary Concept] OR "5-(2-cyclohexylidene-ethyl)-5-ethylbarbiturate"[Supplementary Concept] OR "6-(1H-imidazol-1-yl)-7-nitro-2,3(1H,4H)-quinoxalinedione"[Supplementary Concept] OR "6-methoxytryptoline"[Supplementary Concept] OR "7-nitroindazole"[Supplementary Concept] OR "abecarnil"[Supplementary Concept] OR "Acetazolamide"[mesh] OR "alpha-hexachlorocyclohexane"[Supplementary Concept] OR "angelicin"[Supplementary Concept] OR "anthranilic acid"[Supplementary Concept] OR "bemethyl"[Supplementary Concept] OR "benzobarbital"[Supplementary Concept] OR "bretazenil"[Supplementary Concept] OR "brivaracetam"[Supplementary Concept] OR "Bromides"[mesh] OR "Cannabidiol"[mesh] OR "Carbamazepine"[mesh] OR "Cenobamate"[Supplementary Concept] OR "CGP 39551"[Supplementary Concept] OR "chlordesmethyldiazepam"[Supplementary Concept] OR "Chlormethiazole"[mesh] OR "Clobazam"[mesh] OR "Clonazepam"[mesh] OR "Clorazepate Dipotassium"[mesh] OR "denzimol"[Supplementary Concept] OR "deramciclane"[Supplementary Concept] OR "Diazepam"[mesh] OR "Dimethadione"[mesh] OR "dipropylacetamide"[Supplementary Concept] OR "DN 1417"[Supplementary Concept] OR "doramectin"[Supplementary Concept] OR "eperisone"[Supplementary Concept] OR "eslicarbazepine acetate"[Supplementary Concept] OR "Estazolam"[mesh] OR "Ethosuximide"[mesh] OR "ethotoin"[Supplementary Concept] OR "ethylphenylhydantoin"[Supplementary Concept] OR "ezogabine"[Supplementary Concept] OR "Felbamate"[mesh] OR "fludiazepam"[Supplementary Concept] OR "Flunarizine"[mesh] OR "fosphenytoin"[Supplementary Concept] OR "Gabapentin"[mesh] OR "gaboxadol"[Supplementary Concept] OR "gidazepam"[Supplementary Concept] OR "glutamic acid diethyl ester"[Supplementary Concept] OR "GYKI 52466"[Supplementary Concept] OR "indeloxazine"[Supplementary Concept] OR "indol-3-yl pyruvic acid"[Supplementary Concept] OR "kavain"[Supplementary Concept] OR "L 701324"[Supplementary Concept] OR "Lacosamide"[mesh] OR "Lamotrigine"[mesh] OR "Levetiracetam"[mesh] OR "Lorazepam"[mesh] OR "loreclezole"[Supplementary Concept] OR "Magnesium Sulfate"[mesh] OR "mebeverine"[Supplementary Concept] OR "Medazepam"[mesh] OR "Mephenytoin"[mesh] OR "Mephobarbital"[mesh] OR "Meprobamate"[mesh] OR "methsuximide"[Supplementary Concept] OR "milacemide"[Supplementary Concept] OR "N-(4,4-diphenyl-3-butenyl)nipecotic acid"[Supplementary Concept] OR "N-desmethylclobazam"[Supplementary Concept] OR "NCS 382"[Supplementary Concept] OR "neo-kyotorphin"[Supplementary Concept] OR "neurotropin"[Supplementary Concept] OR "nimetazepam"[Supplementary Concept] OR "Nitrazepam"[mesh] OR "NNC 711"[Supplementary Concept] OR "Org 2766"[Supplementary Concept] OR "Oxcarbazepine"[mesh] OR "padsevonil"[Supplementary Concept] OR "Paraldehyde"[mesh] OR "PD 117302"[Supplementary Concept] OR "phenazepam"[Supplementary Concept] OR "pheneturide"[Supplementary Concept] OR "Phenobarbital"[mesh] OR "Phenytoin"[mesh] OR "pipequaline"[Supplementary Concept] OR "Pregabalin"[mesh] OR "Primidone"[mesh] OR "progabide"[Supplementary Concept] OR "progabide acid"[Supplementary Concept] OR "remacemide"[Supplementary Concept] OR "Riluzole"[mesh] OR "rimcazole"[Supplementary Concept] OR "rufinamide"[Supplementary Concept] OR "ryodipine"[Supplementary Concept] OR "sidnocarb"[Supplementary Concept] OR "stiripentol"[Supplementary Concept] OR "sulthiame"[Supplementary Concept] OR "taglutimide"[Supplementary Concept] OR "Thiopental"[mesh] OR "thioperamide"[Supplementary Concept] OR "Tiagabine"[mesh] OR "Tiletamine"[mesh] OR "tizanidine"[Supplementary Concept] OR "Topiramate"[mesh] OR "tramiprosate"[Supplementary Concept] OR "Trimethadione"[mesh] OR "U 54494A"[Supplementary Concept] OR "Valproic Acid"[mesh] OR "vanillin"[Supplementary Concept] OR "Vigabatrin"[mesh] OR "zaleplon"[Supplementary Concept] OR "ZK 91296"[Supplementary Concept] OR "ZK 93423"[Supplementary Concept] OR "ZK 93426"[Supplementary Concept] OR "Zonisamide"[mesh] OR "1,3-ditolylguanidine"[tw] OR "1-(4-chlorophenyl)-4-piperidin-1-yl-1,5-dihydroimidazol-2-one"[tw] OR "2,3-dioxo-6-nitro-7-sulfamoylbenzo(f)quinoxaline"[tw] OR "2,3-piperidinedicarboxylic acid"[tw] OR "2-(2,3-dicarboxycyclopropyl)glycine"[tw] OR "2-amino-4-methyl-5-phosphono-3-pentenoic acid"[tw] OR "2-amino-4-phosphonobutyric acid"[tw] OR "2-amino-7-phosphonoheptanoic acid"[tw] OR "2-fluoro-2-phenyl-1,3-propanediyl dicarbamate"[tw] OR "2-propyl-2-pentenoic acid"[tw] OR "3-(2-carboxypiperazin-4-yl)propyl-1-phosphonic acid"[tw] OR "4-amino-3-phenylbutyric acid"[tw] OR "4-phenyl-perhydropyrrole(1,2-a)pyrazine-1,3-dione"[tw] OR "5-(2-cyclohexylidene-ethyl)-5-ethylbarbiturate"[tw] OR "6-(1H-imidazol-1-yl)-7-nitro-2,3(1H,4H)-quinoxalinedione"[tw] OR "6-methoxytryptoline"[tw] OR "7-nitroindazole"[tw] OR "abecarnil"[tw] OR "Acetazolamide"[tw] OR "alpha-hexachlorocyclohexane"[tw] OR "angelicin"[tw] OR "anthranilic acid"[tw] OR "bemethyl"[tw] OR "benzobarbital"[tw] OR "bretazenil"[tw] OR "brivaracetam"[tw] OR "Bromides"[tw] OR "Cannabidiol"[tw] OR "Carbamazepine"[tw] OR "Cenobamate"[tw] OR "CGP 39551"[tw] OR "chlordesmethyldiazepam"[tw] OR "Chlormethiazole"[tw] OR "Clobazam"[tw] OR "Clonazepam"[tw] OR "Clorazepate Dipotassium"[tw] OR "denzimol"[tw] OR "deramciclane"[tw] OR "Diazepam"[tw] OR "Dimethadione"[tw] OR "dipropylacetamide"[tw] OR "DN 1417"[tw] OR "doramectin"[tw] OR "eperisone"[tw] OR "eslicarbazepine acetate"[tw] OR "Estazolam"[tw] OR "Ethosuximide"[tw] OR "ethotoin"[tw] OR "ethylphenylhydantoin"[tw] OR "ezogabine"[tw] OR "Felbamate"[tw] OR "fludiazepam"[tw] OR "Flunarizine"[tw] OR "fosphenytoin"[tw] OR "Gabapentin"[tw] OR "gaboxadol"[tw] OR "gidazepam"[tw] OR "glutamic acid diethyl ester"[tw] OR "GYKI 52466"[tw] OR "indeloxazine"[tw] OR "indol-3-yl pyruvic acid"[tw] OR "kavain"[tw] OR "L 701324"[tw] OR "Lacosamide"[tw] OR "Lamotrigine"[tw] OR "Levetiracetam"[tw] OR "Lorazepam"[tw] OR "loreclezole"[tw] OR "Magnesium Sulfate"[tw] OR "mebeverine"[tw] OR "Medazepam"[tw] OR "Mephenytoin"[tw] OR "Mephobarbital"[tw] OR "Meprobamate"[tw] OR "methsuximide"[tw] OR "milacemide"[tw] OR "N-(4,4-diphenyl-3-butenyl)nipecotic acid"[tw] OR "N-desmethylclobazam"[tw] OR "NCS 382"[tw] OR "neo-kyotorphin"[tw] OR "neurotropin"[tw] OR "nimetazepam"[tw] OR "Nitrazepam"[tw] OR "NNC 711"[tw] OR "Org 2766"[tw] OR "Oxcarbazepine"[tw] OR "padsevonil"[tw] OR "Paraldehyde"[tw] OR "PD 117302"[tw] OR "phenazepam"[tw] OR "pheneturide"[tw] OR "Phenobarbital"[tw] OR "Phenytoin"[tw] OR "pipequaline"[tw] OR "Pregabalin"[tw] OR "Primidone"[tw] OR "progabide"[tw] OR "progabide acid"[tw] OR "remacemide"[tw] OR "Riluzole"[tw] OR "rimcazole"[tw] OR "rufinamide"[tw] OR "ryodipine"[tw] OR "sidnocarb"[tw] OR "stiripentol"[tw] OR "sulthiame"[tw] OR "taglutimide"[tw] OR "Thiopental"[tw] OR "thioperamide"[tw] OR "Tiagabine"[tw] OR "Tiletamine"[tw] OR "tizanidine"[tw] OR "Topiramate"[tw] OR "tramiprosate"[tw] OR "Trimethadione"[tw] OR "U 54494A"[tw] OR "Valproic Acid"[tw] OR "vanillin"[tw] OR "Vigabatrin"[tw] OR "zaleplon"[tw] OR "ZK 91296"[tw] OR "ZK 93423"[tw] OR "ZK 93426"[tw] OR "Zonisamide"[tw]) AND ("Treatment Outcome"[Mesh] OR "Treatment outcome"[tw] OR "Treatment Outcomes"[tw] OR "Clinical Effectiveness"[tw] OR "Clinical Efficacy"[tw] OR "Patient Relevant Outcome"[tw] OR "Patient-Relevant Outcomes"[tw] OR "Treatment Effectiveness"[tw] OR "Treatment Efficacy"[tw] OR "Treatment Failure"[tw] OR "treatment fail"[tw] OR "Treatment Failure"[title/abstract:~3] OR "treatment fail"[title/abstract:~3] OR "treatment fail*"[tw] OR "Outcome Assessment, Health Care"[Mesh] OR "outcome"[tw] OR "outcomes"[tw] OR "outcome*"[tw] OR "effectiveness"[tw] OR "effectivity"[tw] OR "efficacy"[tw] OR "efficac*"[tw] OR "retention rate"[tw] OR "retention rates"[tw] OR "seizure free"[tw] OR "seizure free*"[tw] OR "seizure freedom"[tw] OR "seizure freedom*"[tw] OR "seizure reduction"[tw] OR "seizure reduction*"[tw] OR "seizure decrease"[tw] OR "seizure decreas*"[tw] OR "seizure decline"[tw] OR "seizure decline"[title/abstract:~3] OR "seizure control"[tw] OR "seizure response"[tw] OR "seizure responses"[tw] OR "seizure frequency"[tw] OR "seizure frequencies"[tw] OR "engel scale"[tw] OR "international league against epilepsy"[tw] OR "international league against epilepsy"[title/abstract:~6] OR "ilae"[tw] OR "Disease Progression"[mesh] OR "developing seizure"[title/abstract:~3] OR "developing seizures"[title/abstract:~3] OR "newly developed seizure"[title/abstract:~3] OR "newly developed seizures"[title/abstract:~3] OR "new-onset seizure"[title/abstract:~3] OR "new-onset seizures"[title/abstract:~3] OR "postoperative seizure"[title/abstract:~3] OR "postoperative seizures"[title/abstract:~3] OR "seizure development"[title/abstract:~3] OR "seizure naive"[title/abstract:~3] OR "seizure risk"[title/abstract:~3] OR "seizures development"[title/abstract:~3] OR "seizures naive"[title/abstract:~3] OR "seizures risk"[title/abstract:~3] OR "without seizure"[title/abstract:~3] OR "without seizures"[title/abstract:~3] OR "developing epilepsy"[title/abstract:~3] OR "developing epileptic"[title/abstract:~3] OR "newly developed epilepsy"[title/abstract:~3] OR "newly developed epileptic"[title/abstract:~3] OR "new-onset epilepsy"[title/abstract:~3] OR "new-onset epileptic"[title/abstract:~3] OR "postoperative epilepsy"[title/abstract:~3] OR "postoperative epileptic"[title/abstract:~3] OR "epilepsy development"[title/abstract:~3] OR "epilepsy naive"[title/abstract:~3] OR "epilepsy risk"[title/abstract:~3] OR "epileptic development"[title/abstract:~3] OR "epileptic naive"[title/abstract:~3] OR "epileptic risk"[title/abstract:~3] OR "without epilepsy"[title/abstract:~3] OR "without epileptic"[title/abstract:~3])) **OR** (("Brain Neoplasms/**complications**"[majr:noexp]) AND ("Epilepsy"[majr] OR "Epilepsy"[ti] OR "epileptic"[ti] OR "epilepsia"[ti] OR "epilep*"[ti] OR "Seizures"[majr] OR "seizure"[ti] OR "seizures"[ti] OR "Epilepticus"[ti] OR "insult"[ti] OR "insults"[ti] OR "convulsion"[ti] OR "convulsions"[ti] OR "Anticonvulsants"[majr] OR "Anticonvulsants"[ti] OR "Anticonvulsant"[ti] OR "Antiepileptic"[ti] OR "Antiepileptic"[ti] OR "Antiseizure"[ti] OR "Antiseizures"[ti] OR "1,3-ditolylguanidine"[Supplementary Concept] OR "1-(4-chlorophenyl)-4-piperidin-1-yl-1,5-dihydroimidazol-2-one"[Supplementary Concept] OR "2,3-dioxo-6-nitro-7-sulfamoylbenzo(f)quinoxaline"[Supplementary Concept] OR "2,3-piperidinedicarboxylic acid"[Supplementary Concept] OR "2-(2,3-dicarboxycyclopropyl)glycine"[Supplementary Concept] OR "2-amino-4-methyl-5-phosphono-3-pentenoic acid"[Supplementary Concept] OR "2-amino-4-phosphonobutyric acid"[Supplementary Concept] OR "2-amino-7-phosphonoheptanoic acid"[Supplementary Concept] OR "2-fluoro-2-phenyl-1,3-propanediyl dicarbamate"[Supplementary Concept] OR "2-propyl-2-pentenoic acid"[Supplementary Concept] OR "3-(2-carboxypiperazin-4-yl)propyl-1-phosphonic acid"[Supplementary Concept] OR "4-amino-3-phenylbutyric acid"[Supplementary Concept] OR "4-phenyl-perhydropyrrole(1,2-a)pyrazine-1,3-dione"[Supplementary Concept] OR "5-(2-cyclohexylidene-ethyl)-5-ethylbarbiturate"[Supplementary Concept] OR "6-(1H-imidazol-1-yl)-7-nitro-2,3(1H,4H)-quinoxalinedione"[Supplementary Concept] OR "6-methoxytryptoline"[Supplementary Concept] OR "7-nitroindazole"[Supplementary Concept] OR "abecarnil"[Supplementary Concept] OR "Acetazolamide"[majr] OR "alpha-hexachlorocyclohexane"[Supplementary Concept] OR "angelicin"[Supplementary Concept] OR "anthranilic acid"[Supplementary Concept] OR "bemethyl"[Supplementary Concept] OR "benzobarbital"[Supplementary Concept] OR "bretazenil"[Supplementary Concept] OR "brivaracetam"[Supplementary Concept] OR "Bromides"[majr] OR "Cannabidiol"[majr] OR "Carbamazepine"[majr] OR "Cenobamate"[Supplementary Concept] OR "CGP 39551"[Supplementary Concept] OR "chlordesmethyldiazepam"[Supplementary Concept] OR "Chlormethiazole"[majr] OR "Clobazam"[majr] OR "Clonazepam"[majr] OR "Clorazepate Dipotassium"[majr] OR "denzimol"[Supplementary Concept] OR "deramciclane"[Supplementary Concept] OR "Diazepam"[majr] OR "Dimethadione"[majr] OR "dipropylacetamide"[Supplementary Concept] OR "DN 1417"[Supplementary Concept] OR "doramectin"[Supplementary Concept] OR "eperisone"[Supplementary Concept] OR "eslicarbazepine acetate"[Supplementary Concept] OR "Estazolam"[majr] OR "Ethosuximide"[majr] OR "ethotoin"[Supplementary Concept] OR "ethylphenylhydantoin"[Supplementary Concept] OR "ezogabine"[Supplementary Concept] OR "Felbamate"[majr] OR "fludiazepam"[Supplementary Concept] OR "Flunarizine"[majr] OR "fosphenytoin"[Supplementary Concept] OR "Gabapentin"[majr] OR "gaboxadol"[Supplementary Concept] OR "gidazepam"[Supplementary Concept] OR "glutamic acid diethyl ester"[Supplementary Concept] OR "GYKI 52466"[Supplementary Concept] OR "indeloxazine"[Supplementary Concept] OR "indol-3-yl pyruvic acid"[Supplementary Concept] OR "kavain"[Supplementary Concept] OR "L 701324"[Supplementary Concept] OR "Lacosamide"[majr] OR "Lamotrigine"[majr] OR "Levetiracetam"[majr] OR "Lorazepam"[majr] OR "loreclezole"[Supplementary Concept] OR "Magnesium Sulfate"[majr] OR "mebeverine"[Supplementary Concept] OR "Medazepam"[majr] OR "Mephenytoin"[majr] OR "Mephobarbital"[majr] OR "Meprobamate"[majr] OR "methsuximide"[Supplementary Concept] OR "milacemide"[Supplementary Concept] OR "N-(4,4-diphenyl-3-butenyl)nipecotic acid"[Supplementary Concept] OR "N-desmethylclobazam"[Supplementary Concept] OR "NCS 382"[Supplementary Concept] OR "neo-kyotorphin"[Supplementary Concept] OR "neurotropin"[Supplementary Concept] OR "nimetazepam"[Supplementary Concept] OR "Nitrazepam"[majr] OR "NNC 711"[Supplementary Concept] OR "Org 2766"[Supplementary Concept] OR "Oxcarbazepine"[majr] OR "padsevonil"[Supplementary Concept] OR "Paraldehyde"[majr] OR "PD 117302"[Supplementary Concept] OR "phenazepam"[Supplementary Concept] OR "pheneturide"[Supplementary Concept] OR "Phenobarbital"[majr] OR "Phenytoin"[majr] OR "pipequaline"[Supplementary Concept] OR "Pregabalin"[majr] OR "Primidone"[majr] OR "progabide"[Supplementary Concept] OR "progabide acid"[Supplementary Concept] OR "remacemide"[Supplementary Concept] OR "Riluzole"[majr] OR "rimcazole"[Supplementary Concept] OR "rufinamide"[Supplementary Concept] OR "ryodipine"[Supplementary Concept] OR "sidnocarb"[Supplementary Concept] OR "stiripentol"[Supplementary Concept] OR "sulthiame"[Supplementary Concept] OR "taglutimide"[Supplementary Concept] OR "Thiopental"[majr] OR "thioperamide"[Supplementary Concept] OR "Tiagabine"[majr] OR "Tiletamine"[majr] OR "tizanidine"[Supplementary Concept] OR "Topiramate"[majr] OR "tramiprosate"[Supplementary Concept] OR "Trimethadione"[majr] OR "U 54494A"[Supplementary Concept] OR "Valproic Acid"[majr] OR "vanillin"[Supplementary Concept] OR "Vigabatrin"[majr] OR "zaleplon"[Supplementary Concept] OR "ZK 91296"[Supplementary Concept] OR "ZK 93423"[Supplementary Concept] OR "ZK 93426"[Supplementary Concept] OR "Zonisamide"[majr] OR "1,3-ditolylguanidine"[ti] OR "1-(4-chlorophenyl)-4-piperidin-1-yl-1,5-dihydroimidazol-2-one"[ti] OR "2,3-dioxo-6-nitro-7-sulfamoylbenzo(f)quinoxaline"[ti] OR "2,3-piperidinedicarboxylic acid"[ti] OR "2-(2,3-dicarboxycyclopropyl)glycine"[ti] OR "2-amino-4-methyl-5-phosphono-3-pentenoic acid"[ti] OR "2-amino-4-phosphonobutyric acid"[ti] OR "2-amino-7-phosphonoheptanoic acid"[ti] OR "2-fluoro-2-phenyl-1,3-propanediyl dicarbamate"[ti] OR "2-propyl-2-pentenoic acid"[ti] OR "3-(2-carboxypiperazin-4-yl)propyl-1-phosphonic acid"[ti] OR "4-amino-3-phenylbutyric acid"[ti] OR "4-phenyl-perhydropyrrole(1,2-a)pyrazine-1,3-dione"[ti] OR "5-(2-cyclohexylidene-ethyl)-5-ethylbarbiturate"[ti] OR "6-(1H-imidazol-1-yl)-7-nitro-2,3(1H,4H)-quinoxalinedione"[ti] OR "6-methoxytryptoline"[ti] OR "7-nitroindazole"[ti] OR "abecarnil"[ti] OR "Acetazolamide"[ti] OR "alpha-hexachlorocyclohexane"[ti] OR "angelicin"[ti] OR "anthranilic acid"[ti] OR "bemethyl"[ti] OR "benzobarbital"[ti] OR "bretazenil"[ti] OR "brivaracetam"[ti] OR "Bromides"[ti] OR "Cannabidiol"[ti] OR "Carbamazepine"[ti] OR "Cenobamate"[ti] OR "CGP 39551"[ti] OR "chlordesmethyldiazepam"[ti] OR "Chlormethiazole"[ti] OR "Clobazam"[ti] OR "Clonazepam"[ti] OR "Clorazepate Dipotassium"[ti] OR "denzimol"[ti] OR "deramciclane"[ti] OR "Diazepam"[ti] OR "Dimethadione"[ti] OR "dipropylacetamide"[ti] OR "DN 1417"[ti] OR "doramectin"[ti] OR "eperisone"[ti] OR "eslicarbazepine acetate"[ti] OR "Estazolam"[ti] OR "Ethosuximide"[ti] OR "ethotoin"[ti] OR "ethylphenylhydantoin"[ti] OR "ezogabine"[ti] OR "Felbamate"[ti] OR "fludiazepam"[ti] OR "Flunarizine"[ti] OR "fosphenytoin"[ti] OR "Gabapentin"[ti] OR "gaboxadol"[ti] OR "gidazepam"[ti] OR "glutamic acid diethyl ester"[ti] OR "GYKI 52466"[ti] OR "indeloxazine"[ti] OR "indol-3-yl pyruvic acid"[ti] OR "kavain"[ti] OR "L 701324"[ti] OR "Lacosamide"[ti] OR "Lamotrigine"[ti] OR "Levetiracetam"[ti] OR "Lorazepam"[ti] OR "loreclezole"[ti] OR "Magnesium Sulfate"[ti] OR "mebeverine"[ti] OR "Medazepam"[ti] OR "Mephenytoin"[ti] OR "Mephobarbital"[ti] OR "Meprobamate"[ti] OR "methsuximide"[ti] OR "milacemide"[ti] OR "N-(4,4-diphenyl-3-butenyl)nipecotic acid"[ti] OR "N-desmethylclobazam"[ti] OR "NCS 382"[ti] OR "neo-kyotorphin"[ti] OR "neurotropin"[ti] OR "nimetazepam"[ti] OR "Nitrazepam"[ti] OR "NNC 711"[ti] OR "Org 2766"[ti] OR "Oxcarbazepine"[ti] OR "padsevonil"[ti] OR "Paraldehyde"[ti] OR "PD 117302"[ti] OR "phenazepam"[ti] OR "pheneturide"[ti] OR "Phenobarbital"[ti] OR "Phenytoin"[ti] OR "pipequaline"[ti] OR "Pregabalin"[ti] OR "Primidone"[ti] OR "progabide"[ti] OR "progabide acid"[ti] OR "remacemide"[ti] OR "Riluzole"[ti] OR "rimcazole"[ti] OR "rufinamide"[ti] OR "ryodipine"[ti] OR "sidnocarb"[ti] OR "stiripentol"[ti] OR "sulthiame"[ti] OR "taglutimide"[ti] OR "Thiopental"[ti] OR "thioperamide"[ti] OR "Tiagabine"[ti] OR "Tiletamine"[ti] OR "tizanidine"[ti] OR "Topiramate"[ti] OR "tramiprosate"[ti] OR "Trimethadione"[ti] OR "U 54494A"[ti] OR "Valproic Acid"[ti] OR "vanillin"[ti] OR "Vigabatrin"[ti] OR "zaleplon"[ti] OR "ZK 91296"[ti] OR "ZK 93423"[ti] OR "ZK 93426"[ti] OR "Zonisamide"[ti]) AND ("Treatment Outcome"[Mesh] OR "Treatment outcome"[tw] OR "Treatment Outcomes"[tw] OR "Clinical Effectiveness"[tw] OR "Clinical Efficacy"[tw] OR "Patient Relevant Outcome"[tw] OR "Patient-Relevant Outcomes"[tw] OR "Treatment Effectiveness"[tw] OR "Treatment Efficacy"[tw] OR "Treatment Failure"[tw] OR "treatment fail"[tw] OR "Treatment Failure"[title/abstract:~3] OR "treatment fail"[title/abstract:~3] OR "treatment fail*"[tw] OR "Outcome Assessment, Health Care"[Mesh] OR "outcome"[tw] OR "outcomes"[tw] OR "outcome*"[tw] OR "effectiveness"[tw] OR "effectivity"[tw] OR "efficacy"[tw] OR "efficac*"[tw] OR "retention rate"[tw] OR "retention rates"[tw] OR "seizure free"[tw] OR "seizure free*"[tw] OR "seizure freedom"[tw] OR "seizure freedom*"[tw] OR "seizure reduction"[tw] OR "seizure reduction*"[tw] OR "seizure decrease"[tw] OR "seizure decreas*"[tw] OR "seizure decline"[tw] OR "seizure decline"[title/abstract:~3] OR "seizure control"[tw] OR "seizure response"[tw] OR "seizure responses"[tw] OR "seizure frequency"[tw] OR "seizure frequencies"[tw] OR "engel scale"[tw] OR "international league against epilepsy"[tw] OR "international league against epilepsy"[title/abstract:~6] OR "ilae"[tw] OR "Disease Progression"[mesh] OR "developing seizure"[title/abstract:~3] OR "developing seizures"[title/abstract:~3] OR "newly developed seizure"[title/abstract:~3] OR "newly developed seizures"[title/abstract:~3] OR "new-onset seizure"[title/abstract:~3] OR "new-onset seizures"[title/abstract:~3] OR "postoperative seizure"[title/abstract:~3] OR "postoperative seizures"[title/abstract:~3] OR "seizure development"[title/abstract:~3] OR "seizure naive"[title/abstract:~3] OR "seizure risk"[title/abstract:~3] OR "seizures development"[title/abstract:~3] OR "seizures naive"[title/abstract:~3] OR "seizures risk"[title/abstract:~3] OR "without seizure"[title/abstract:~3] OR "without seizures"[title/abstract:~3] OR "developing epilepsy"[title/abstract:~3] OR "developing epileptic"[title/abstract:~3] OR "newly developed epilepsy"[title/abstract:~3] OR "newly developed epileptic"[title/abstract:~3] OR "new-onset epilepsy"[title/abstract:~3] OR "new-onset epileptic"[title/abstract:~3] OR "postoperative epilepsy"[title/abstract:~3] OR "postoperative epileptic"[title/abstract:~3] OR "epilepsy development"[title/abstract:~3] OR "epilepsy naive"[title/abstract:~3] OR "epilepsy risk"[title/abstract:~3] OR "epileptic development"[title/abstract:~3] OR "epileptic naive"[title/abstract:~3] OR "epileptic risk"[title/abstract:~3] OR "without epilepsy"[title/abstract:~3] OR "without epileptic"[title/abstract:~3])) **OR** (("Brain tumor"[ti] OR "brain tumors"[ti] OR "brain tumour"[ti] OR "brain tumours"[ti] OR "brain neoplasm"[ti] OR "brain neoplasms"[ti] OR "central nervous system cancer"[ti] OR "cns cancer"[ti] OR "cns cancers"[ti] OR "cerebral cancer*"[ti] OR "intracerebral cancer*"[ti] OR "intracranial cancer*"[ti] OR "cranial cancer*"[ti] OR "central nervous system tumor*"[ti] OR "central nervous system tumour"[ti] OR "tumor of the central nervous system"[ti] OR "tumour of the central nervous system"[ti] OR "cns tumor"[ti] OR "cns tumors"[ti] OR "cns tumour"[ti] OR "cns tumours"[ti] OR "cerebral tumor"[ti] OR "cerebral tumour"[ti] OR "cerebral tumors"[ti] OR "cerebral tumours"[ti] OR "intracerebral tumor"[ti] OR "intracerebral tumour"[ti] OR "intracerebral tumors"[ti] OR "intracerebral tumours"[ti] OR "intracranial tumor"[ti] OR "intracranial tumour"[ti] OR "intracranial tumors"[ti] OR "intracranial tumours"[ti] OR "cranial tumor"[ti] OR "cranial tumour"[ti] OR "cranial tumors"[ti] OR "cranial tumours"[ti] OR "brain malignancy"[ti] OR "brain malignancies"[ti] OR "cns malignancy"[ti] OR "malignancy of the central nervous system"[title:~3] OR "malignancies of the central nervous system"[title:~3] OR "malignant central nervous system"[title:~3] OR "malignancy central nervous system"[title:~3] OR "malignancies central nervous system"[title:~3] OR "malignant central nervous system"[title:~3] OR "central nervous system malignancy"[ti] OR "central nervous system malignancies"[ti] OR "cerebral malignancy"[ti] OR "cerebral malignancies"[ti] OR "intracerebral malignancy"[ti] OR "intracerebral malignancies"[ti] OR "intracranial malignancy"[ti] OR "intracranial malignancies"[ti] OR "cranial malignancy"[ti] OR "cranial malignancies"[ti] OR "brain carcinoma"[ti] OR "carcinoma of the brain"[ti] OR "brain carcinomas"[ti] OR "cns carcinoma"[ti] OR "cns carcinomas"[ti] OR "carcinoma of the central nervous system"[title:~3] OR "carcinomas of the central nervous system"[title:~3] OR "carcinoma central nervous system"[title:~3] OR "carcinomas central nervous system"[title:~3] OR "central nervous system carcinoma"[ti] OR "central nervous system carcinomas"[ti] OR "cerebral carcinoma"[ti] OR "cerebral carcinomas"[ti] OR "intracerebral carcinoma"[ti] OR "intracerebral carcinomas"[ti] OR "intracranial carcinoma"[ti] OR "intracranial carcinomas"[ti] OR "cranial carcinoma"[ti] OR "cranial carcinomas"[ti]) AND ("Epilepsy"[majr] OR "Epilepsy"[ti] OR "epileptic"[ti] OR "epilepsia"[ti] OR "epilep*"[ti] OR "Seizures"[majr] OR "seizure"[ti] OR "seizures"[ti] OR "Epilepticus"[ti] OR "insult"[ti] OR "insults"[ti] OR "convulsion"[ti] OR "convulsions"[ti] OR "Anticonvulsants"[majr] OR "Anticonvulsants"[ti] OR "Anticonvulsant"[ti] OR "Antiepileptic"[ti] OR "Antiepileptic"[ti] OR "Antiseizure"[ti] OR "Antiseizures"[ti] OR "1,3-ditolylguanidine"[Supplementary Concept] OR "1-(4-chlorophenyl)-4-piperidin-1-yl-1,5-dihydroimidazol-2-one"[Supplementary Concept] OR "2,3-dioxo-6-nitro-7-sulfamoylbenzo(f)quinoxaline"[Supplementary Concept] OR "2,3-piperidinedicarboxylic acid"[Supplementary Concept] OR "2-(2,3-dicarboxycyclopropyl)glycine"[Supplementary Concept] OR "2-amino-4-methyl-5-phosphono-3-pentenoic acid"[Supplementary Concept] OR "2-amino-4-phosphonobutyric acid"[Supplementary Concept] OR "2-amino-7-phosphonoheptanoic acid"[Supplementary Concept] OR "2-fluoro-2-phenyl-1,3-propanediyl dicarbamate"[Supplementary Concept] OR "2-propyl-2-pentenoic acid"[Supplementary Concept] OR "3-(2-carboxypiperazin-4-yl)propyl-1-phosphonic acid"[Supplementary Concept] OR "4-amino-3-phenylbutyric acid"[Supplementary Concept] OR "4-phenyl-perhydropyrrole(1,2-a)pyrazine-1,3-dione"[Supplementary Concept] OR "5-(2-cyclohexylidene-ethyl)-5-ethylbarbiturate"[Supplementary Concept] OR "6-(1H-imidazol-1-yl)-7-nitro-2,3(1H,4H)-quinoxalinedione"[Supplementary Concept] OR "6-methoxytryptoline"[Supplementary Concept] OR "7-nitroindazole"[Supplementary Concept] OR "abecarnil"[Supplementary Concept] OR "Acetazolamide"[majr] OR "alpha-hexachlorocyclohexane"[Supplementary Concept] OR "angelicin"[Supplementary Concept] OR "anthranilic acid"[Supplementary Concept] OR "bemethyl"[Supplementary Concept] OR "benzobarbital"[Supplementary Concept] OR "bretazenil"[Supplementary Concept] OR "brivaracetam"[Supplementary Concept] OR "Bromides"[majr] OR "Cannabidiol"[majr] OR "Carbamazepine"[majr] OR "Cenobamate"[Supplementary Concept] OR "CGP 39551"[Supplementary Concept] OR "chlordesmethyldiazepam"[Supplementary Concept] OR "Chlormethiazole"[majr] OR "Clobazam"[majr] OR "Clonazepam"[majr] OR "Clorazepate Dipotassium"[majr] OR "denzimol"[Supplementary Concept] OR "deramciclane"[Supplementary Concept] OR "Diazepam"[majr] OR "Dimethadione"[majr] OR "dipropylacetamide"[Supplementary Concept] OR "DN 1417"[Supplementary Concept] OR "doramectin"[Supplementary Concept] OR "eperisone"[Supplementary Concept] OR "eslicarbazepine acetate"[Supplementary Concept] OR "Estazolam"[majr] OR "Ethosuximide"[majr] OR "ethotoin"[Supplementary Concept] OR "ethylphenylhydantoin"[Supplementary Concept] OR "ezogabine"[Supplementary Concept] OR "Felbamate"[majr] OR "fludiazepam"[Supplementary Concept] OR "Flunarizine"[majr] OR "fosphenytoin"[Supplementary Concept] OR "Gabapentin"[majr] OR "gaboxadol"[Supplementary Concept] OR "gidazepam"[Supplementary Concept] OR "glutamic acid diethyl ester"[Supplementary Concept] OR "GYKI 52466"[Supplementary Concept] OR "indeloxazine"[Supplementary Concept] OR "indol-3-yl pyruvic acid"[Supplementary Concept] OR "kavain"[Supplementary Concept] OR "L 701324"[Supplementary Concept] OR "Lacosamide"[majr] OR "Lamotrigine"[majr] OR "Levetiracetam"[majr] OR "Lorazepam"[majr] OR "loreclezole"[Supplementary Concept] OR "Magnesium Sulfate"[majr] OR "mebeverine"[Supplementary Concept] OR "Medazepam"[majr] OR "Mephenytoin"[majr] OR "Mephobarbital"[majr] OR "Meprobamate"[majr] OR "methsuximide"[Supplementary Concept] OR "milacemide"[Supplementary Concept] OR "N-(4,4-diphenyl-3-butenyl)nipecotic acid"[Supplementary Concept] OR "N-desmethylclobazam"[Supplementary Concept] OR "NCS 382"[Supplementary Concept] OR "neo-kyotorphin"[Supplementary Concept] OR "neurotropin"[Supplementary Concept] OR "nimetazepam"[Supplementary Concept] OR "Nitrazepam"[majr] OR "NNC 711"[Supplementary Concept] OR "Org 2766"[Supplementary Concept] OR "Oxcarbazepine"[majr] OR "padsevonil"[Supplementary Concept] OR "Paraldehyde"[majr] OR "PD 117302"[Supplementary Concept] OR "phenazepam"[Supplementary Concept] OR "pheneturide"[Supplementary Concept] OR "Phenobarbital"[majr] OR "Phenytoin"[majr] OR "pipequaline"[Supplementary Concept] OR "Pregabalin"[majr] OR "Primidone"[majr] OR "progabide"[Supplementary Concept] OR "progabide acid"[Supplementary Concept] OR "remacemide"[Supplementary Concept] OR "Riluzole"[majr] OR "rimcazole"[Supplementary Concept] OR "rufinamide"[Supplementary Concept] OR "ryodipine"[Supplementary Concept] OR "sidnocarb"[Supplementary Concept] OR "stiripentol"[Supplementary Concept] OR "sulthiame"[Supplementary Concept] OR "taglutimide"[Supplementary Concept] OR "Thiopental"[majr] OR "thioperamide"[Supplementary Concept] OR "Tiagabine"[majr] OR "Tiletamine"[majr] OR "tizanidine"[Supplementary Concept] OR "Topiramate"[majr] OR "tramiprosate"[Supplementary Concept] OR "Trimethadione"[majr] OR "U 54494A"[Supplementary Concept] OR "Valproic Acid"[majr] OR "vanillin"[Supplementary Concept] OR "Vigabatrin"[majr] OR "zaleplon"[Supplementary Concept] OR "ZK 91296"[Supplementary Concept] OR "ZK 93423"[Supplementary Concept] OR "ZK 93426"[Supplementary Concept] OR "Zonisamide"[majr] OR "1,3-ditolylguanidine"[ti] OR "1-(4-chlorophenyl)-4-piperidin-1-yl-1,5-dihydroimidazol-2-one"[ti] OR "2,3-dioxo-6-nitro-7-sulfamoylbenzo(f)quinoxaline"[ti] OR "2,3-piperidinedicarboxylic acid"[ti] OR "2-(2,3-dicarboxycyclopropyl)glycine"[ti] OR "2-amino-4-methyl-5-phosphono-3-pentenoic acid"[ti] OR "2-amino-4-phosphonobutyric acid"[ti] OR "2-amino-7-phosphonoheptanoic acid"[ti] OR "2-fluoro-2-phenyl-1,3-propanediyl dicarbamate"[ti] OR "2-propyl-2-pentenoic acid"[ti] OR "3-(2-carboxypiperazin-4-yl)propyl-1-phosphonic acid"[ti] OR "4-amino-3-phenylbutyric acid"[ti] OR "4-phenyl-perhydropyrrole(1,2-a)pyrazine-1,3-dione"[ti] OR "5-(2-cyclohexylidene-ethyl)-5-ethylbarbiturate"[ti] OR "6-(1H-imidazol-1-yl)-7-nitro-2,3(1H,4H)-quinoxalinedione"[ti] OR "6-methoxytryptoline"[ti] OR "7-nitroindazole"[ti] OR "abecarnil"[ti] OR "Acetazolamide"[ti] OR "alpha-hexachlorocyclohexane"[ti] OR "angelicin"[ti] OR "anthranilic acid"[ti] OR "bemethyl"[ti] OR "benzobarbital"[ti] OR "bretazenil"[ti] OR "brivaracetam"[ti] OR "Bromides"[ti] OR "Cannabidiol"[ti] OR "Carbamazepine"[ti] OR "Cenobamate"[ti] OR "CGP 39551"[ti] OR "chlordesmethyldiazepam"[ti] OR "Chlormethiazole"[ti] OR "Clobazam"[ti] OR "Clonazepam"[ti] OR "Clorazepate Dipotassium"[ti] OR "denzimol"[ti] OR "deramciclane"[ti] OR "Diazepam"[ti] OR "Dimethadione"[ti] OR "dipropylacetamide"[ti] OR "DN 1417"[ti] OR "doramectin"[ti] OR "eperisone"[ti] OR "eslicarbazepine acetate"[ti] OR "Estazolam"[ti] OR "Ethosuximide"[ti] OR "ethotoin"[ti] OR "ethylphenylhydantoin"[ti] OR "ezogabine"[ti] OR "Felbamate"[ti] OR "fludiazepam"[ti] OR "Flunarizine"[ti] OR "fosphenytoin"[ti] OR "Gabapentin"[ti] OR "gaboxadol"[ti] OR "gidazepam"[ti] OR "glutamic acid diethyl ester"[ti] OR "GYKI 52466"[ti] OR "indeloxazine"[ti] OR "indol-3-yl pyruvic acid"[ti] OR "kavain"[ti] OR "L 701324"[ti] OR "Lacosamide"[ti] OR "Lamotrigine"[ti] OR "Levetiracetam"[ti] OR "Lorazepam"[ti] OR "loreclezole"[ti] OR "Magnesium Sulfate"[ti] OR "mebeverine"[ti] OR "Medazepam"[ti] OR "Mephenytoin"[ti] OR "Mephobarbital"[ti] OR "Meprobamate"[ti] OR "methsuximide"[ti] OR "milacemide"[ti] OR "N-(4,4-diphenyl-3-butenyl)nipecotic acid"[ti] OR "N-desmethylclobazam"[ti] OR "NCS 382"[ti] OR "neo-kyotorphin"[ti] OR "neurotropin"[ti] OR "nimetazepam"[ti] OR "Nitrazepam"[ti] OR "NNC 711"[ti] OR "Org 2766"[ti] OR "Oxcarbazepine"[ti] OR "padsevonil"[ti] OR "Paraldehyde"[ti] OR "PD 117302"[ti] OR "phenazepam"[ti] OR "pheneturide"[ti] OR "Phenobarbital"[ti] OR "Phenytoin"[ti] OR "pipequaline"[ti] OR "Pregabalin"[ti] OR "Primidone"[ti] OR "progabide"[ti] OR "progabide acid"[ti] OR "remacemide"[ti] OR "Riluzole"[ti] OR "rimcazole"[ti] OR "rufinamide"[ti] OR "ryodipine"[ti] OR "sidnocarb"[ti] OR "stiripentol"[ti] OR "sulthiame"[ti] OR "taglutimide"[ti] OR "Thiopental"[ti] OR "thioperamide"[ti] OR "Tiagabine"[ti] OR "Tiletamine"[ti] OR "tizanidine"[ti] OR "Topiramate"[ti] OR "tramiprosate"[ti] OR "Trimethadione"[ti] OR "U 54494A"[ti] OR "Valproic Acid"[ti] OR "vanillin"[ti] OR "Vigabatrin"[ti] OR "zaleplon"[ti] OR "ZK 91296"[ti] OR "ZK 93423"[ti] OR "ZK 93426"[ti] OR "Zonisamide"[ti]) AND ("Treatment Outcome"[Mesh] OR "Treatment outcome"[tw] OR "Treatment Outcomes"[tw] OR "Clinical Effectiveness"[tw] OR "Clinical Efficacy"[tw] OR "Patient Relevant Outcome"[tw] OR "Patient-Relevant Outcomes"[tw] OR "Treatment Effectiveness"[tw] OR "Treatment Efficacy"[tw] OR "Treatment Failure"[tw] OR "treatment fail"[tw] OR "Treatment Failure"[title/abstract:~3] OR "treatment fail"[title/abstract:~3] OR "treatment fail*"[tw] OR "Outcome Assessment, Health Care"[Mesh] OR "outcome"[tw] OR "outcomes"[tw] OR "outcome*"[tw] OR "effectiveness"[tw] OR "effectivity"[tw] OR "efficacy"[tw] OR "efficac*"[tw] OR "retention rate"[tw] OR "retention rates"[tw] OR "seizure free"[tw] OR "seizure free*"[tw] OR "seizure freedom"[tw] OR "seizure freedom*"[tw] OR "seizure reduction"[tw] OR "seizure reduction*"[tw] OR "seizure decrease"[tw] OR "seizure decreas*"[tw] OR "seizure decline"[tw] OR "seizure decline"[title/abstract:~3] OR "seizure control"[tw] OR "seizure response"[tw] OR "seizure responses"[tw] OR "seizure frequency"[tw] OR "seizure frequencies"[tw] OR "engel scale"[tw] OR "international league against epilepsy"[tw] OR "international league against epilepsy"[title/abstract:~6] OR "ilae"[tw] OR "Disease Progression"[mesh] OR "developing seizure"[title/abstract:~3] OR "developing seizures"[title/abstract:~3] OR "newly developed seizure"[title/abstract:~3] OR "newly developed seizures"[title/abstract:~3] OR "new-onset seizure"[title/abstract:~3] OR "new-onset seizures"[title/abstract:~3] OR "postoperative seizure"[title/abstract:~3] OR "postoperative seizures"[title/abstract:~3] OR "seizure development"[title/abstract:~3] OR "seizure naive"[title/abstract:~3] OR "seizure risk"[title/abstract:~3] OR "seizures development"[title/abstract:~3] OR "seizures naive"[title/abstract:~3] OR "seizures risk"[title/abstract:~3] OR "without seizure"[title/abstract:~3] OR "without seizures"[title/abstract:~3] OR "developing epilepsy"[title/abstract:~3] OR "developing epileptic"[title/abstract:~3] OR "newly developed epilepsy"[title/abstract:~3] OR "newly developed epileptic"[title/abstract:~3] OR "new-onset epilepsy"[title/abstract:~3] OR "new-onset epileptic"[title/abstract:~3] OR "postoperative epilepsy"[title/abstract:~3] OR "postoperative epileptic"[title/abstract:~3] OR "epilepsy development"[title/abstract:~3] OR "epilepsy naive"[title/abstract:~3] OR "epilepsy risk"[title/abstract:~3] OR "epileptic development"[title/abstract:~3] OR "epileptic naive"[title/abstract:~3] OR "epileptic risk"[title/abstract:~3] OR "without epilepsy"[title/abstract:~3] OR "without epileptic"[title/abstract:~3]))**)** NOT (("Infant"[mesh] OR "infant"[ti] OR "infants"[ti] OR "Child"[mesh] OR "child"[ti] OR "children"[ti] OR "Adolescent"[mesh] OR "adolescent"[ti] OR "adolescents"[ti] OR "pediatric"[ti] OR "paediatric"[ti] OR "pediatric*"[ti] OR "paediatric*"[ti]) NOT ("Adult"[mesh] OR "adult"[ti] OR "adults"[ti] OR "middle aged"[ti] OR "elderly"[ti])) NOT (("review"[pt] OR "review"[ti]) AND ("0000/01/01"[PDAT] : "2014/12/31"[PDAT]))

**Supplementary 2: detailed quality assessment of the Risk Of Bias In Non-randomized Studies of Interventions tool**

1. *Potential confounding domains and important co-interventions*

Confounding domains***

- Age
- Sex
- Primary tumor type
- BM location
- Number of BMs
- Size of largest BM
- KPS
- Intratumoral hemorrhage

Co-interventions

- Surgical resection
- Stereotactic radiosurgery
- Whole-brain radiotherapy
- Chemotherapy
- Immune therapy or targeted therapy
- Corticosteroids
- Antiseizure medication (ASM)

*Type of epileptic seizures (focal or generalized) was not included as a confounder. In neuro-oncology, most of the time a broad ASM such as levetiracetam is prescribed, and the choice is not based on the type of epileptic seizure. Van der Meer et al.^1^ showed that the type of ASMs are similar between patients with focal and generalized epileptic seizures.

1. *Evaluation of bias per study*

| **Domains** | **Risk of bias** | **Reason** |
| --- | --- | --- |
| **Bahna (2022)** | | |
| Confounding | Critical | Many potential confounding factors were identified (e.g. preoperative KPS). Due to sample size limitations, Cox regression analysis was not performed to investigate the correlation between potential confounders and seizure outcomes. |
| Selection of patients | Serious | Only patients with at least 3 months of follow-up of seizures were included in the analysis, which might have caused immortal time bias. Start of intervention and follow-up coincided. |
| Classification of interventions | Low | The intervention was clearly defined. Classification of intervention was based on information from medical records documented at the time of the intervention. The intervention had been performed at the hospital which also collected the data. |
| Deviations from intended interventions | Serious | ASM use was standardized for all included patients. Other important co-interventions (e.g. radiotherapy and systemic therapy) were not considered in the study. |
| Missing data | Moderate | The number of deaths during follow-up was not mentioned in the article and death was not included in the analyses (i.e., by competing risks analyses). As median overall survival was not significantly different between patients with a favorable and unfavorable seizure outcome, this might have biased results of the incidence of seizures only marginally. |
| Measurement of outcomes | Moderate | Seizure outcomes were clearly defined and were extracted from medical records. Patients and the treating physicians who recorded the outcomes were aware of the patients’ assigned intervention during the intervention. |
| Selection of reported results | Low | All patients who received the intervention between a certain time period were included in the study. Outcome measures were clearly defined and analyses were internally consistent between Methods and Results in the paper. |
| Overall risk of bias | Critical | Critical risk of bias with confounding, serious risk of bias with selection of patients, serious risk of bias with deviations from intended interventions, moderate risk of bias with missing data, and moderate risk of bias with measurement of outcomes. Therefore, overall risk of bias is classified as critical risk of bias. |

| **Domains** | **Risk of bias** | **Reason** |
| --- | --- | --- |
| **Garcia (2022)** | | |
| Confounding | Serious | Many potential confounding factors were identified (e.g. number of BM) and included in univariable and multivariable analysis. Some variables (e.g. preoperative KPS as a measure of clinical performance) were not considered. |
| Selection of patients | Low | All patients who met the inclusion criteria and received the intervention within a certain time period were included in the study. Start of intervention and follow-up coincided. |
| Classification of interventions | Low | The intervention was clearly defined. Classification of intervention was based on information from medical records documented at the time of the intervention. The intervention had been performed at the hospital which also collected the data. |
| Deviations from intended interventions | Moderate | ASM use was standardized for patients with preoperative seizures. Other important co-interventions (prior radiotherapy and systemic checkpoint inhibitor use) were included in univariable and multivariable analysis. Use of corticosteroids and chemotherapy were not considered in the study. |
| Missing data | Serious | The number of deaths during follow-up was not mentioned in the article and death was not included in the analyses (i.e., by competing risks analyses). This could have biased results of the incidence of seizures. |
| Measurement of outcomes | Moderate | Seizure outcomes were clearly defined and were extracted from medical records. Patients and the treating physicians who recorded the outcomes were aware of the patients’ assigned intervention during the intervention. |
| Selection of reported results | Low | All patients who received the intervention with a certain time period were included in the study. Outcome measures were clearly defined and analyses were internally consistent between Methods and Results in the paper. |
| Overall risk of bias | Serious | Serious risk of bias with confounding, moderate risk of deviations from intended interventions, serious risk of bias with missing data, and moderate risk of bias with measurement of outcomes. Therefore, overall risk of bias is classified as serious risk of bias. |

| **Domains** | **Risk of bias** | **Reason** |
| --- | --- | --- |
| **Puri (2020)** | | |
| Confounding | Serious | Many potential confounding factors were identified (e.g. number of BM) and included in univariable and multivariable analysis. Stratification was performed for age and gender. Some potential confounders (e.g. intratumoral hemorrhage) were not considered. |
| Selection of patients | Low | All patients who met the inclusion criteria and received the intervention within a certain time period were included in the study. Start of intervention and follow-up coincided. |
| Classification of interventions | Low | The intervention was clearly defined. Classification of intervention was based on information from medical records documented at the time of the intervention. The intervention had been performed at the hospital which also collected the data. |
| Deviations from intended interventions | Critical | No relevant co-interventions were considered in the study at all. |
| Missing data | Serious | The number of deaths during follow-up was not mentioned in the article and death was not included in the analyses (i.e., by competing risks analyses). This could have biased results of the incidence of seizures. |
| Measurement of outcomes | Moderate | Seizure outcomes were clearly defined and were extracted from medical records. Patients and the treating physicians who recorded the outcomes were aware of the patients’ assigned intervention during the intervention. |
| Selection of reported results | Low | All patients who received the intervention with a certain time period were included in the study. Outcome measures were clearly defined and analyses were internally consistent between Methods and Results in the paper. |
| Overall risk of bias | Critical | Serious risk of bias with confounding, critical risk of deviations from intended interventions, serious risk of bias with missing data, and moderate risk of bias with measurement of outcomes. Therefore, overall risk of bias is classified as critical risk of bias. |

| **Domains** | **Risk of bias** | **Reason** |
| --- | --- | --- |
| **Wu (2017)** | | |
| Confounding | Serious | Many potential confounding factors were identified (e.g. size of BM) and included in univariable and multivariable analysis. Some important variables (e.g. intratumoral hemorrhage) were not considered. |
| Selection of patients | Low | All patients who met the inclusion criteria and had received the intervention within a certain time period were included in the study. Start of intervention and follow-up coincided. |
| Classification of interventions | Low | The intervention was clearly defined. Classification of intervention was based on information from medical records documented at the time of the intervention. The intervention had been performed at the hospital which also collected the data. |
| Deviations from intended interventions | Moderate | Most important co-interventions (e.g. ASMs and postoperative radiotherapy) were included in univariable and multivariable analysis. Only the use of corticosteroids was not assessed in the study. |
| Missing data | Serious | Deaths were censored by Cox regression. Although patient numbers considerably lowered during follow-up time, no competing risk analysis was performed to take the risk of death into account. This could have biased results of the incidence of seizures. |
| Measurement of outcomes | Moderate | Seizure outcomes were clearly defined and were extracted from medical records. Patients and the treating physicians who recorded the outcomes were aware of the patients’ assigned intervention during the intervention. |
| Selection of reported results | Low | All patients who received the intervention with a certain time period were included in the study. Outcome measures were clearly defined and analyses were internally consistent between Methods and Results in the paper. |
| Overall risk of bias | Serious | Serious risk of bias with confounding, moderate risk of deviations from intended interventions, serious risk of bias with missing data and moderate risk of bias with measurement of outcomes. Therefore, overall risk of bias is classified as serious risk of bias. |

| **Domains** | **Risk of bias** | **Reason** |
| --- | --- | --- |
| **Sanmillan (2017)** | | |
| Confounding | Critical | The sample size was small (n=13) and no control group was included. No statistical analyses were performed to correct seizure outcomes for potential confounding factors. |
| Selection of patients | Low | All patients who met the inclusion criteria and had received the intervention within a certain time period were included in the study. Start of intervention and follow-up coincided. |
| Classification of interventions | Low | The intervention was clearly defined. Classification of intervention was based on information from medical records documented at the time of the intervention. The intervention had been performed at the hospital which also collected the data. |
| Deviations from intended interventions | Moderate | All patients with seizures used ASMs pre- and postoperative. Conform the institution’s protocol, all patients who were fit enough received WBRT after surgical resection. The use of corticosteroids was not considered. |
| Missing data | Serious | Although 6 patients died during the follow-up, no competing risk analysis was performed to take the risk of death into account. This could have biased results of the incidence of seizures. |
| Measurement of outcomes | No information | It is unclear how seizure outcomes were collected. |
| Selection of reported results | Low | All patients who received the intervention with a certain time period were included in the study. Outcome measures were clearly defined and analyses were internally consistent between Methods and Results in the paper. |
| Overall risk of bias | Critical | Critical risk of bias with confounding, moderate risk of deviations from intended interventions, serious risk of bias with missing data, and no information to rule out bias with measurement of outcomes. Therefore, overall risk of bias is classified as critical risk of bias. |

| **Domains** | **Risk of bias** | **Reason** |
| --- | --- | --- |
| **Pelletier (2021)** | | |
| Confounding | Serious | The included BM patients were matched with a control group of high-grade glioma patients based on six different potential confounders. However, some potential confounders were not considered (e.g. intratumoral hemorrhage) and the sample size was small (n=10). |
| Selection of patients | Low | All patients who met the inclusion criteria and had received the intervention within a certain time period were included in the study. Start of intervention and follow-up coincided. |
| Classification of interventions | Low | The intervention was clearly defined. Classification of intervention was based on information from medical records documented at the time of the intervention. The intervention had been performed at the hospital which also collected the data. |
| Deviations from intended interventions | Serious | Intervention and control group were different regarding two important co-interventions (preoperative radiotherapy and chemotherapy). Use of other co-interventions was not assessed (e.g. ASMs). |
| Missing data | Low | Data were reasonably complete. Outcomes (including deaths) were clearly reported in a table for each individual patient. |
| Measurement of outcomes | No information | It is unclear how seizure outcomes were collected and how seizure control was defined. |
| Selection of reported results | Low | All patients who received the intervention with a certain time period were included in the study. Outcome measures were clearly defined and analyses were internally consistent between Methods and Results in the paper. |
| Overall risk of bias | Serious | Serious risk of bias with confounding, serious risk of deviations from intended interventions and no information to rule out bias with measurement of outcomes. Therefore, overall risk of bias is classified as serious risk of bias. |

| **Domains** | **Risk of bias** | **Reason** |
| --- | --- | --- |
| **Huntoon (2023)** | | |
| Confounding | Serious | Some potential confounding factors were identified (e.g. location of BM) and included in univariable analysis to test the association with seizures at initial presentation, but no multivariable analysis was performed in case of significant correlations. Some important variables (e.g. intratumoral hemorrhage) were not considered. No association with seizure outcomes after surgery was tested. |
| Selection of patients | Serious | Patients with certain origins of BM with a low number of seizures were excluded from the final analysis, which differed from the described inclusion and exclusion criteria. Details regarding the follow-up period were not provided. Based on the fact that all patients with seizure outcomes had a follow-up of ≥2 years, immortal time bias have likely occurred. |
| Classification of interventions | Low | The intervention was clearly defined. Classification of intervention was based on information from medical records documented at the time of the intervention. The intervention had been performed at the hospital which also collected the data. |
| Deviations from intended interventions | Critical | Patients were assigned to the surgical resection or nonsurgical resection group, but no details on any other treatments received in both groups were not provided. |
| Missing data | Serious | Despite significant survival differences between the surgery and no surgery groups in Kaplan-Meier analysis, no competing risk analysis was performed to take the risk of death into account. This could have biased results of the incidence of seizures. |
| Measurement of outcomes | Moderate | Seizure outcomes were clearly defined and were extracted from medical records. Patients and the treating physicians who recorded the outcomes were aware of the patients’ assigned intervention during the intervention. |
| Selection of reported results | Serious | Patients with certain origins of BM were excluded from the study (see also ‘Selection of patients’). Subgroup analyses for melanoma patients were described in the article, which were not described in the Methods section of the paper. |
| Overall risk of bias | Critical | Serious risk of bias with confounding, serious risk of bias with selection of patients, critical risk of deviations from intended interventions, serious risk of bias with missing data, moderate risk of bias with measurement of outcomes, and serious risk of selection of reported results. Therefore, overall risk of bias is classified as critical risk of bias. |

| **Domains** | **Risk of bias** | **Reason** |
| --- | --- | --- |
| **Cummins (2023)** | | |
| Confounding | Serious | Some potential confounding factors were identified (e.g. location of BM) and included in univariable and multivariable analysis. One important variable (KPS) was not considered. |
| Selection of patients | Serious | All patients who met the inclusion criteria and had received the intervention within a certain time period were included in the study. Seizure outcomes were measured after the intervention starting after hospital discharge to exclude seizures which might have been related to the surgery itself, which might have caused immortal time bias. |
| Classification of interventions | Low | The intervention was clearly defined. Classification of intervention was based on information from medical records documented at the time of the intervention. The intervention had been performed at the hospital which also collected the data. |
| Deviations from intended interventions | Moderate | Use of ASMs pre- and postoperatively was standardized. Most other important co-interventions (e.g. postoperative WBRT and SRT) were included in univariable and multivariable analysis. Use of corticosteroids was not assessed in the study. |
| Missing data | Serious | Deaths were censored by Cox regression. However, no competing risk analysis was performed to take the risk of death into account. This could have biased results of the incidence of seizures. |
| Measurement of outcomes | Moderate | Seizure outcomes were clearly defined and were extracted from medical records. Patients and the treating physicians who recorded the outcomes were aware of the patients’ assigned intervention during the intervention. |
| Selection of reported results | Low | All patients who received the intervention with a certain time period were included in the study. Outcome measures were clearly defined and analyses were internally consistent between Methods and Results in the paper. |
| Overall risk of bias | Serious | Serious risk of bias with confounding, serious risk of bias with selection of patients, moderate risk of deviations from intended interventions, serious risk of bias with missing data, and moderate risk of bias with measurement of outcomes. Therefore, overall risk of bias is classified as serious risk of bias. |

| **Domains** | **Risk of bias** | **Reason** |
| --- | --- | --- |
| **Lee (2013)** | | |
| Confounding | Critical | No statistical analyses were performed to correct seizure outcomes for potential confounding factors. |
| Selection of patients | Low | All patients who met the inclusion criteria and had received the intervention within a certain time period were included in the study. Start of intervention and follow-up coincided. |
| Classification of interventions | Low | The intervention was clearly defined. Classification of intervention was based on information from medical records documented at the time of the intervention. The intervention had been performed at the hospital which also collected the data. |
| Deviations from intended interventions | Serious | All patients were treated with ASMs for a median of 30 days after an epileptic seizure. Patients’ use of some other important co-interventions (surgical resection and WBRT) was described in the article, although statistical analyses were not sufficient to accurately determine the impact of different co-interventions on seizure outcomes. |
| Missing data | Serious | 2 of 32 patients (6%) with BTRE were lost to follow-up, which was less than in the group of patients without BTRE. 7 patients with BTRE died during the follow-up period. No competing risk analysis was performed to take the risk of death into account. This could have biased results of the incidence of seizures. |
| Measurement of outcomes | Moderate | Seizure outcomes were extracted from medical records. Patients and the treating physicians who recorded the outcomes were aware of the patients’ assigned intervention during the intervention. |
| Selection of reported results | Low | All patients who received the intervention with a certain time period were included in the study. Outcome measures were clearly defined and analyses were internally consistent between Methods and Results in the paper. |
| Overall risk of bias | Critical | Critical risk of bias with confounding, serious risk of deviations from intended interventions, serious risk of bias with missing data, and moderate risk of bias with measurement of outcomes. Therefore, overall risk of bias is classified as critical risk of bias. |

| **Domains** | **Risk of bias** | **Reason** |
| --- | --- | --- |
| **Borgelt (1980)*** | | |
| Confounding | Serious | We are aware that the risk of bias for the domain confounding cannot be assessed optimally in a single-arm clinical trial. Due to the differences in performance status between the two studies, results were presented for both studies separately. Some other potential confounders were assessed in the study (e.g. primary tumor type), but others were not (e.g. intratumoral hemorrhage). |
| Selection of patients | No information | It was unclear how patients were recruited to receive WBRT during the identified time frame and if all consecutive patients meeting the inclusion criteria were entered in the study. Start of intervention and follow-up coincided. |
| Classification of interventions | Low | The intervention was clearly defined. Classification of intervention was based on information documented at the time of the intervention. |
| Deviations from intended interventions | Serious | The implementation of the intervention was successful. Patients were excluded from the study if ‘new specific anti-cancer treatment’ was administered within two weeks prior the start of the study. Patients’ use of two important co-interventions (chemotherapy and corticosteroids) was described in the article but not included in regression analysis, whereas use of other important co-interventions (e.g. use of ASMs and surgical resection) was not considered. |
| Missing data | Serious | 84% of patients completed the planned course of treatment and 6% died during treatment, which means that 10% was lost to follow-up, which is considered a reasonable rate. Based on the presented results, the authors stated that time to neurologic progression (the primary outcome) was highly dependent on survival, but no competing risk analysis was performed to take the risk of death into account in primary outcomes or seizure outcomes. This could have biased results of the incidence of seizures. |
| Measurement of outcomes | Moderate | Seizure outcomes were recorded for the study specifically, but the method of recording was unclear. Patients and people delivering the intervention were aware that patients received WBRT. |
| Selection of reported results | Moderate | The selection of patients into the study was unclear. No a priori research plan could be found. Outcome measures were clearly defined and analyses were internally consistent between Methods and Results in the paper. |
| Overall risk of bias | Serious | Serious risk of bias with confounding, serious risk of deviations from intended interventions, serious risk of bias with missing data, moderate risk bias with of measurements of outcomes, moderate risk of bias with selection of reported results, and too little information to rule out selection bias. Therefore, overall risk of bias is classified as serious risk of bias. |

*The article by Borgelt et al. (1980) contained 2 studies with identical study designs and at identical risk of bias, which were therefore discussed as one here.

| **Domains** | **Risk of bias** | **Reason** |
| --- | --- | --- |
| **Miller (2023)** | | |
| Confounding | Serious | Some potential confounding factors were identified (e.g. number of BM) and included in univariable and multivariable analysis. Other important variables (e.g. KPS) were not considered. |
| Selection of patients | Serious | All patients who met the inclusion criteria and had received the intervention within a certain time period were included in the study. Patients with a follow-up of less than 3 months were excluded from the analyses, which has likely caused immortal time bias. |
| Classification of interventions | Low | The intervention was clearly defined. Classification of intervention was based on information from medical records documented at the time of the intervention. The intervention had been performed at the hospital which also collected the data. |
| Deviations from intended interventions | Moderate | Some important co-interventions (surgery and ASM use) were included in univariable and multivariable analysis. Two important co-interventions (use of corticosteroids and systemic therapy) were not assessed in the study. |
| Missing data | Serious | With Cox regression, authors determined that seizure incidence did not impact overall survival, although no hazard ratio was provided. No competing risk analysis was performed to take the risk of death into account in seizure outcomes. This could have biased results of the incidence of seizures. |
| Measurement of outcomes | Moderate | Seizure outcomes were clearly defined and were extracted from medical records. Patients and the treating physicians who recorded the outcomes were aware of the patients’ assigned intervention during the intervention. |
| Selection of reported results | Low | All patients who received the intervention with a certain time period were included in the study. Outcome measures were clearly defined, and analyses were internally consistent between Methods and Results in the paper. |
| Overall risk of bias | Serious | Serious risk of bias with confounding, serious risk of bias with selection of patients, moderate risk of deviations from intended interventions, serious risk of bias with missing data, and moderate risk of bias with measurement of outcomes. Therefore, overall risk of bias is classified as serious risk of bias. |

| **Domains** | **Risk of bias** | **Reason** |
| --- | --- | --- |
| **Kim (2011)** | | |
| Confounding | Serious | Patients were selected based on one potential confounding variable (number of BM) and other potential confounding factors were identified and included in univariable and multivariable analysis to determine the association with seizure free survival (e.g. location of BM). One variable (intratumoral hemorrhage) was not assessed. |
| Selection of patients | Low | All patients who met the inclusion criteria were included in the study. Start of intervention and follow-up coincided. |
| Classification of interventions | Low | The intervention was clearly defined. Classification of intervention was based on information collected at the time of the intervention. |
| Deviations from intended interventions | Moderate | Use of topiramate during chemotherapy was standardized. Regression analysis was performed to explore the effect of two important co-interventions (radiotherapy versus surgery) on seizure free survival. The effect of two co-interventions (corticosteroids and systemic treatments) on seizure outcomes was not considered. |
| Missing data | Serious | In analyses of seizure-free survival, deaths were censored by Cox regression. However, no competing risk analysis was performed to take the risk of death into account. This could have biased results of the incidence of seizures. |
| Measurement of outcomes | Moderate | Seizure outcomes were clearly defined. To record the primary outcomes, patients had monthly check-ups and were asked to contact the researchers in case of seizures. Patients and the people delivering the interventions were aware of the patients’ assigned intervention during the intervention. |
| Selection of reported results | Low | All patients who received the intervention with a certain time period were included in the study. Outcome measures were clearly defined and analyses were internally consistent between Methods and Results in the paper. |
| Overall risk of bias | Serious | Serious risk of bias with confounding, moderate risk of deviations from intended interventions, serious risk of bias with missing data, and moderate risk of bias with measurement of outcomes. Therefore, overall risk of bias is classified as serious risk of bias. |

| **Domains** | **Risk of bias** | **Reason** |
| --- | --- | --- |
| **Newton (2007)** | | |
| Confounding | Critical | No statistical analyses were performed to correct seizure outcomes for potential confounding factors. |
| Selection of patients | Serious | The authors stated that all patients who had received the intervention in one center were included in the study, although the time window of patient inclusion was unclear. Patients with less than 1 month of follow-up were excluded, which might have caused immortal time bias. Start of intervention and follow-up coincided. |
| Classification of interventions | Low | The intervention was clearly defined. Classification of intervention was based on information collected at the time of the intervention. The intervention and data collection had been performed at the same hospital. |
| Deviations from intended interventions | Critical | The effect of co-interventions on seizure outcomes was not considered, although patients likely received co-interventions. |
| Missing data | Low | Data were reasonably complete. As all patients were alive when seizure outcomes were measured (at 1 month), no correction for death was necessary. |
| Measurement of outcomes | Moderate | Seizure outcomes were clearly defined and were extracted from medical records. Patients and the treating physicians who recorded the outcomes were aware of the patients’ assigned intervention during the intervention. |
| Selection of reported results | Low | All patients who received the intervention with a certain time period were included in the study. Outcome measures were clearly defined, and analyses were internally consistent between Methods and Results in the paper. |
| Overall risk of bias | Critical | Critical risk of bias with confounding, serious risk of bias with selection of patients, critical risk of deviations from intended interventions, and moderate risk of bias with measurement of outcomes. Therefore, overall risk of bias is classified as critical risk of bias. |

| **Domains** | **Risk of bias** | **Reason** |
| --- | --- | --- |
| **Maschio (2022)** | | |
| Confounding | Serious | Some potential confounding factors (e.g. BM location) were identified and included in univariable and multivariable analysis to determine the association with efficacy of ASM, although no effect sizes of the results were provided (only p-values). Some variables (e.g. number of BM and intratumoral hemorrhage) were not assessed. |
| Selection of patients | Low | All patients who met the inclusion criteria and had received the intervention within a certain time period were selected for included in the study. Start of intervention and follow-up coincided. |
| Classification of interventions | Low | The intervention was clearly defined. Classification of intervention was based on information collected at the time of the intervention. The intervention and data collection had been performed at the same hospital. |
| Deviations from intended interventions | Moderate | The effect of co-interventions on seizure outcomes was analyzed using stratification, although no effect measures of the results were provided (only p-values). Presence of one co-intervention (corticosteroids) was not considered. |
| Missing data | Critical | 69 of 111 patients selected for inclusion dropped out, a part of those because of “poor compliance”. Poor compliance to ASM might have been related to adverse effects/low effectiveness of ASMs. In the analysis of time to treatment failure, deaths were censored by Cox regression. However, no competing risk analysis was performed to take the risk of death into account. This could have biased results of the incidence of seizures. |
| Measurement of outcomes | Moderate | Seizure outcomes were clearly defined and were extracted from medical records. Patients and the treating physicians who recorded the outcomes were aware of the patients’ assigned intervention during the intervention. |
| Selection of reported results | Low | All patients who received the intervention with a certain time period were included in the study. Outcome measures were clearly defined and analyses were internally consistent between Methods and Results in the paper. |
| Overall risk of bias | Critical | Serious risk of bias with confounding, moderate risk of deviations from intended interventions, critical risk of bias with missing data, and moderate risk of bias with measurement of outcomes. Therefore, overall risk of bias is classified as critical risk of bias. |

| **Domains** | **Risk of bias** | **Reason** |
| --- | --- | --- |
| **Maschio (2010)** | | |
| Confounding | Serious | Potential confounding by some variables (e.g. KPS) was analyzed. Some factors (e.g. intratumoral hemorrhage) were not taken into account. |
| Selection of patients | Low | All patients who met the inclusion criteria and had received the intervention within a certain time period were selected for included in the study. Start of intervention and follow-up coincided. |
| Classification of interventions | Low | The intervention was clearly defined. Classification of intervention was based on information collected at the time of the intervention. |
| Deviations from intended interventions | Serious | The effect of some co-interventions (chemotherapy and/or radiotherapy) on seizure outcomes was analyzed using logistic regression, although no effect measures of the results were provided (only p-values). Presence of other co-interventions (surgical resection and corticosteroids) was not considered. |
| Missing data | Critical | 18 of 48 patients selected for inclusion dropped out, because they never returned to the research center. It was unclear if the dropout was related to adverse effects/low effectiveness of ASMs. Logistic regression instead of Cox regression was used to analyze the impact of systemic treatments on seizure control, and no competing risk analysis was performed to take the risk of death into account. This could have biased results of the incidence of seizures. |
| Measurement of outcomes | Moderate | Seizure outcomes were clearly defined and to collect them, patients used seizure diaries and had monthly check-ups. Patients and the treating physicians who recorded the outcomes were aware of the patients’ assigned intervention during the intervention. |
| Selection of reported results | Low | All patients who received the intervention with a certain time period were included in the study. Outcome measures were clearly defined, and analyses were internally consistent between Methods and Results in the paper. |
| Overall risk of bias | Critical | Serious risk of bias with confounding, serious risk of deviations from intended interventions, critical risk of bias with missing data, and moderate risk of bias with measurement of outcomes. Therefore, overall risk of bias is classified as critical risk of bias. |

**Supplementary 3: Seizure outcomes at 6 months postoperatively**

|  | **At 6 months** | | |
| --- | --- | --- | --- |
| **Article** | **N** | **Seizure freedom, n** | **%** |
| Wu 2017 | 41 | 36 | 88 |
| Garcia 2022 | 84 | 48 | 57 |
| Cummins 2023 | 26 | 13 | 50 |
| **Total** | 151 | WA | 64% |

WA=weighted average

**Supplementary 4:** **Seizure outcomes at 3 and 6 months postoperatively,
including articles at critical overall risk of bias**

| **Article** | **Risk of bias** | **At 3 months** | | | **At 6 months** | | |
| --- | --- | --- | --- | --- | --- | --- | --- |
|  |  | **N** | **Seizure freedom, n** | **%** | **N** | **Seizure freedom, n** | **%** |
| Bahna 2022 | Critical | 38 | 34 | 90 |  |  |  |
| Puri 2020 | Critical | 48 | 36 | 75 |  |  |  |
| Wu 2017 | Serious | 59 | 52 | 88 | 41 | 36 | 88 |
| Garcia 2022 | Serious |  |  |  | 84 | 48 | 57 |
| Sanmillan 2017 | Critical |  |  |  | 13 | 13 | 100 |
| Cummins 2023 | Serious |  |  |  | 26 | 13 | 50 |
| **Total** |  | 145 | WA | 84% | 164 | WA | 67% |

WA=weighted average
